# Two modes of Cue2-mediated mRNA cleavage with distinct substrate recognition initiate no-go decay

**DOI:** 10.1093/nar/gkac1172

**Published:** 2022-12-30

**Authors:** Shota Tomomatsu, Atsuya Watanabe, Petr Tesina, Satoshi Hashimoto, Ken Ikeuchi, Sihan Li, Yoshitaka Matsuo, Roland Beckmann, Toshifumi Inada

**Affiliations:** Division of RNA and Gene Regulation, Institute of Medical Science, The University of Tokyo, Minato-Ku 108-8639, Japan; Graduate School of Pharmaceutical Sciences, The University of Tokyo, Bunkyo-Ku, Tokyo, Japan; Graduate School of Pharmaceutical Sciences, Tohoku University, Sendai 980-8578, Japan; Graduate School of Pharmaceutical Sciences, Tohoku University, Sendai 980-8578, Japan; Gene Center and Department of Biochemistry, Feodor-Lynen-Strasse 25, University of Munich, D-81377 Munich, Germany; Graduate School of Pharmaceutical Sciences, Tohoku University, Sendai 980-8578, Japan; Graduate School of Pharmaceutical Sciences, Tohoku University, Sendai 980-8578, Japan; Gene Center and Department of Biochemistry, Feodor-Lynen-Strasse 25, University of Munich, D-81377 Munich, Germany; Division of RNA and Gene Regulation, Institute of Medical Science, The University of Tokyo, Minato-Ku 108-8639, Japan; Graduate School of Pharmaceutical Sciences, Tohoku University, Sendai 980-8578, Japan; Division of RNA and Gene Regulation, Institute of Medical Science, The University of Tokyo, Minato-Ku 108-8639, Japan; Graduate School of Pharmaceutical Sciences, Tohoku University, Sendai 980-8578, Japan; Gene Center and Department of Biochemistry, Feodor-Lynen-Strasse 25, University of Munich, D-81377 Munich, Germany; Division of RNA and Gene Regulation, Institute of Medical Science, The University of Tokyo, Minato-Ku 108-8639, Japan; Graduate School of Pharmaceutical Sciences, Tohoku University, Sendai 980-8578, Japan

## Abstract

Ribosome collisions are recognized by E3 ubiquitin ligase Hel2/ZNF598, leading to RQC (ribosome-associated quality control) and to endonucleolytic cleavage and degradation of the mRNA termed NGD (no-go decay). NGD in yeast requires the Cue2 endonuclease and occurs in two modes, either coupled to RQC (NGD^RQC+^) or RQC uncoupled (NGD^RQC−^). This is mediated by an unknown mechanism of substrate recognition by Cue2. Here, we show that the ubiquitin binding activity of Cue2 is required for NGD^RQC−^ but not for NGD^RQC+^, and that it involves the first two N-terminal Cue domains. In contrast, Trp122 of Cue2 is crucial for NGD^RQC+^. Moreover, Mbf1 is required for quality controls by preventing +1 ribosome frameshifting induced by a rare codon staller. We propose that in Cue2-dependent cleavage upstream of the collided ribosomes (NGD^RQC−^), polyubiquitination of eS7 is recognized by two N-terminal Cue domains of Cue2. In contrast, for the cleavage within collided ribosomes (NGD^RQC+^), the UBA domain, Trp122 and the interaction between Mbf1 and uS3 are critical.

## INTRODUCTION

Ribosome stalling triggers quality control pathways targeting the mRNA (NGD: no-go decay) and the nascent polypeptide (RQC: ribosome-associated quality control). The NGD mRNA quality control pathway occurs when ribosomes are blocked by stable RNA secondary structures, rare codons or polybasic-encoding mRNA stretches (1–8), and it is initiated by an endonucleolytic cleavage of mRNA proximal to the ribosomal stalling site (3). This cleavage results in the production of 5′-NGD and 3′-NGD mRNA intermediates, which are further degraded by the Xrn1 and the exosome exoribonucleases, respectively (3).

In such a stalling event, ribosomal collision that involves a leading stalled ribosome and a following colliding ribosome(s) also triggers the RQC pathway (2,8–14). In a first step, the collided ribosomes are ubiquitinated by the E3 ubiquitin ligase Hel2 in yeast *Saccharomyces cerevisiae* (ZNF598 in mammals) and the E2 enzyme Ubc4 (9,12–14). In the next step, initiation of the RQC pathway requires the RQC-Trigger (RQT) complex which is composed of the RNA helicase Slh1(Rqt2), the ubiquitin-binding protein Cue3 (Rqt3) and the Rqt4 protein (12,14,15). The RQT complex associates with the collided ribosomes and here the ATPase activity of Slh1 and the ubiquitin binding activity of Cue3 have been shown to be responsible for the subunit dissociation of polyubiquitinated stalled ribosomes (12,14). Similarly in the mammalian system, ZNF598 ubiquitinates ribosomal proteins uS10 and eS10 to trigger the subunit dissociation of stalled ribosomes by the homolog of the RQT complex, suggesting a conserved role for ribosome ubiquitination in quality control (10,16–20).

Collided ribosomes stalled on a rare codon stalling sequence are subjected to the NGD pathway which can be dissected into two branches, depending on the coupling with RQC (13). The NGD coupled with RQC is referred to as the NGD^RQC+^, and this pathway induces the endonucleolytic cleavage close to the stall site within the mRNA stretch occupied by the collided ribosomes. NGD^RQC+^ depends on Hel2-mediated K63-linked polyubiquitination of uS10 and on the Slh1-mediated subunit dissociation of the stalling ribosomes (13). In the absence of the subunit dissociation (RQT activity) that depends on the uS10 ubiquitination, RQC is not initiated and the endonucleolytic mRNA cleavages occur upstream of the collision site. This alternative NGD pathway is referred to as the NGD^RQC−^ and, in contrast to NGD^RQC+^, the cleavages in NGD^RQC−^ require K63-linked polyubiquitination of ribosomal protein eS7 via a two-step mechanism. In this process, the E3 ligase Not4 first monoubiquitinates eS7 which is then followed by Hel2-mediated polyubiquitination (13). Together, these findings indicate the dual role of Hel2 leading to two distinct NGD pathways, which require specific ubiquitination events on the collided ribosomes.

Ribosomes which have collided on the endogenous *SDD1* stalling sequence are also subjected to RQC (12). A cryo-electron microscopy (cryo-EM) structure of the ribosome stalled on the *SDD1* mRNA revealed that *SDD1* employs a concerted mechanism for translational stalling, in which the nascent polypeptide chain-induced interference with the peptidyl-transferase center (PTC) is combined with a difficult to decode mRNA for cooperative stalling (12). *In vitro* translation of *SDD1* mRNA enabled the reconstitution of Hel2-dependent polyubiquitination of collided ribosomes and, subsequently, the dissociation of the first stalled polyubiquitinated ribosome in an ATP-dependent manner by the Slh1 helicase subunit of the RQT complex (12). However, it is still unknown whether the NGD is also induced by the *SDD1* staller.

A genetic screen in *S. cerevisiae* identified Cue2 as the primary endonuclease initiating NGD (21). Ribosome profiling and biochemical assays suggest that Cue2 cleaves mRNAs in the A site of collided ribosomes in the presence of Slh1 (21). In *Caenorhabditis elegans*, a homolog of Cue2, NONU-1, was found which is required for mRNA cleavage in the vicinity of collided ribosomes (22), showing the evolutionarily conserved function of NONU-1 and Cue2. Cue2 contains three CUE domains and one UBA domain, and these ubiquitin-binding domains may be responsible for the recognition of ubiquitinated ribosomes that are substrates for NGD since, as mentioned above, Hel2-mediated ribosome ubiquitination is required both for canonical NGD (NGD^RQC+^) and RQC-uncoupled NGD outside the collided ribosomes (NGD^RQC−^). It remains unknown, however, how Cue2 can specifically recognize substrates in two distinct NGD pathways, which require diverse ubiquitination events on the collided ribosomes.

An additional player in the context of ribosome collisions is the multiprotein bridging factor 1 (Mbf1) which is associated with the colliding ribosome and acts with ribosomal proteins Rps3/uS3 and Asc1/RACK1 to prevent frameshifting at inhibitory CGA–CGA codon pairs in yeast (23,24). It has been proposed that Mbf1 maintains the reading frame during the translation of rare codons. Cryo-EM analyses of Mbf1 in the CGACGG-collided ribosomes revealed a conserved 40S ribosomal subunit-binding site at the mRNA entry channel near the collision interface in the rare codons staller (25,26). It has been proposed that ribosome collisions induce +1 ribosome frameshifting (RFS) because the colliding ribosome exerts a pulling force on the mRNA during translocation that promotes slippage of the mRNA, especially on poly(A) tracts (27–29). In this regard, Mbf1 would suppress the +1 RFS on the leading ribosome by binding to the colliding ribosome and locking its 40S subunit to prevent mRNA slippage.

In this study, we performed domain analysis of Cue2 in NGD induced by the rare codons staller. We found that the two N-terminal ubiquitin-binding CUE domains of Cue2 play an important role in the NGD. Specifically, these two CUE domains are required for endonucleolytic mRNA cleavage upstream of the ribosome collision site (NGD^RQC−^) but they are dispensable for the ribosome splitting-dependent mRNA cleavage within the ribosome-covered collision site (NGD^RQC+^). In the case of mRNA cleavage within the ribosome collision site (NGD^RQC+^), the Trp122 residue of Cue2 is specifically required. Moreover, the collision sensor protein Mbf1 associates with colliding ribosomes in the endogenous *SDD1* staller and we found that its interaction with ribosomal protein uS3 is also crucial for NGD^RQC+^. We also found that its interaction with ribosomal protein uS3 is crucial for RQC and NGD^RQC+^ by preventing the +1 RFS. We thus propose that the two distinct ribosome ubiquitination processes in the two modes of NGD result in different modes of Cue2 substrate recognition and activity. In the NGD^RQC+^, uS10 polyubiquitination drives the RQT-mediated subunit dissociation of the collided ribosomes. Given that NGD^RQC+^ depends on the RQT-mediated subunit dissociation of the leading stalled ribosome, we propose that Cue2 may be recruited to the colliding ribosome to cleave the mRNA. In contrast, in NGD^RQC−^, ubiquitination of eS7 is required for the recruitment of Cue2 that cleaves mRNA upstream of the collided ribosomes.

## MATERIALS AND METHODS

### Yeast strains

Yeast strains are listed in Supplementary Table S1. *Saccharomyces cerevisiae* W303-1a-based strains were obtained by established recombination techniques using polymerase chain reaction (PCR)-amplified cassette sequences (*kanMX4*, *hphMX4*, *natMX4*, *natNT2* or *HISMX6*) (30,31). To construct strains carrying essential ribosomal protein genes (uS10 and uS3), the shuffle strain transformed with plasmids expressing mutant ribosomal protein products was grown on an SDC plate containing 0.5 mg/ml 5-fluoro-orotic acid (5-FOA, #F9001-5, Zymo Research, Irvine, CA, USA), and strains lacking URA3 were isolated.

### Plasmids

Plasmids used in this study are listed in Supplementary Table S2. DNA cloning was performed by PCR amplification with gene-specific primers using PrimeSTAR HS DNA polymerase (#R010A, Takara-bio, Shiga, Japan) and T4 DNA ligase (#M0202S, NEB, Ipswich, MA, USA) or a Gibson assembly system. All cloned DNAs amplified by PCR were verified by sequencing.

### Yeast culture and media

Yeast cells were cultured with yeast peptone dextrose (YPD) or synthetic complete (SC) medium with 2% glucose at 30°C and harvested at log phase by centrifugation after discarding the medium. Cell pellets were frozen in liquid nitrogen immediately after harvest and stored at –80°C until used.

### RNA isolation

Total RNA solutions were prepared using the acidic phenol RNA extraction method as follows: the cell pellet was resuspended with 300 μl of RNA buffer [300 mM NaCl, 20 mM Tris–HCl pH 7.5, 10 mM EDTA, 1% sodium dodecylsulfate (SDS), dissolved in diethylpyrocarbonate (DEPC)-treated MilliQ water at room temperature] on ice, followed by immediate addition of 300 μl of water-saturated phenol and vortex mixing for 10 s. The mixture was incubated at 65°C for 5 min, mixed by vortex for 10 s and chilled on ice for 5 min. After centrifugation at 16 000 *g* for 5 min at room temperature, 300 μl of the water layer was transferred to a new 1.5 ml RNase-free tube. A 300 μl aliquot of water-saturated phenol/chloroform/isoamyl alcohol (25:24:1) was added, followed by a 10 s vortex and centrifugation at 16 000 *g* for 5 min at room temperature. Then, 250 μl of the water layer was transferred to a new 1.5 ml RNase-free tube and subjected to ethanol precipitation. The RNA pellet was finally dissolved with 20–30 μl of DEPC-treated water to obtain a total RNA solution.

### RNA electrophoresis and northern blotting

A volume of 6 μl (2 μg) of total RNA solution was mixed with 24 μl of glyoxal mix [600 μl of dimethylsulfoxide (DMSO), 200 μl of deionized 40% glyoxal, 120 μl of 10× MOPS buffer (200 mM MOPS, 50 mM NaOAc, 10 mM EDTA pH 7.0), 62.5 μl of 80% glycerol and 17.5 μl of DEPC-treated water in 1 ml] and 3 μl of RNA loading buffer (50% glycerol, 10 mM EDTA pH 8.0, 0.05% bromophenol blue and 0.05% xylene cyanol). The mixture was incubated at 74°C for 10 min, followed by incubation on ice for 10 min to obtain RNA samples. Aliquots of 7 μl of each sample were electrophoresed at 200 V for 60 min on a 2.0% agarose gel in 1× MOPS buffer (20 mM MOPS, 5 mM NaOAc, 1 mM EDTA pH 7.0), followed by transfer of RNA to Hybond-N+ membranes (GE Healthcare, Chicago, IL, USA) with 20× SSC (3 M NaCl and 300 mM trisodium citrate dihydrate) for 18 h using a capillary system. RNA was cross-linked on the membrane using a CL-1000 ultraviolet cross-linker (UVP) at 120 mJ/cm^2^. The membrane was then incubated with DIG Easy Hyb Granules (#11796895001, Roche) for 1 h in a hybridization oven at 50°C. A digoxigenin (DIG)-labeled probe prepared using the PCR DIG Probe Synthesis Kit (#11636090910, Roche) and DIG-labeled FLAG oligonucleotide (Fasmac) was added and incubated for >18 h, followed by two washes with wash buffer I (2× SSC, 0.1% SDS) for 15 min in a hybridization oven at 50°C, and an additional wash with wash buffer II (0.1× SSC, 0.1% SDS) for 15 min at 50°C. The membrane was then washed with 1× maleic acid buffer (100 mM maleic acid, 150 mM NaCl pH 7.0, adjusted with NaOH) for 10 min at room temperature and incubated with Blocking Reagent (#11096176001, Roche) for 30 min. Anti-digoxigenin-AP, Fab fragments (#11093274910, Roche) were added to the Blocking Reagent, and the membrane was further incubated for 1 h. After that, the membrane was washed with wash buffer III (1× maleic acid buffer, 0.3% Tween-20) for 10 min three times, and equilibrated with equilibration buffer (100 mM Tris–HCl, 100 mM NaCl pH 9.5). The membrane was reacted with CDP-star (#11759051001, Roche) for 10 min, and chemiluminescence was detected with the LAS-4000 system (GE Healthcare). Relative RNA levels were determined using Multi Gauge v3.0 (Fujifilm, Japan).

### Trichloroacetic acid (TCA) precipitation for protein preparation

The yeast cell pellet in a 1.5 ml tube (on ice) was resuspended with 500 μl of ice-cold TCA buffer [20 mM Tris–HCl pH 8.0, 50 mM NH_4_OAc, 2 mM EDTA and 1 mM phenylmethylsulfonyl fluoride (PMSF)] and transferred to a new 1.5 ml tube containing 500 μl of 20% TCA and 500 μl of 0.5 mm Zirconia/Silica beads (BioSpec Products, Bartlesville, OK, USA). The cells were vortexed for 30 s three times, and the supernatant was transferred to a new 1.5 ml tube. Another 500 μl of ice-cold TCA buffer was added to a beads-containing 1.5 ml tube, vortexed for 30 s and then the supernatant was transferred to a 1.5 ml tube. After centrifugation of lysates (14 000 rpm, 15 min, 4°C), the supernatant was discarded, and the pellet was dissolved in sodium dodecylsulfate (SDS) sample buffer [125 mM Tris–HCl pH 6.8, 4% SDS, 20% glycerol, 100 mM dithiothreitol (DTT), 0.01% bromophenol blue; 1500 μl/6OD_600_ in Figures 2C and 4B, Supplementary Figure S1B–D and 150 μl/6OD_600_ in the other figures) and heated at 100°C for 10 min. The protein solution was used for SDS–polyacrylamide gel electrophoresis (PAGE).

### Electrophoresis and western blotting

Protein samples were separated by SDS–PAGE and transferred onto polyvinyldifluoridene (PVDF) membranes (Immobilon-P, Merck Millipore, MA, USA). Membranes were blocked with 5% skim milk in PBST (10 mM Na_2_HPO_4_/NaH_2_PO_4_ pH 7.5, 0.9% NaCl and 0.1% Tween-20), incubated with primary antibodies for 1 h at room temperature, washed three times in PBST and incubated with horseradish peroxidase (HRP)-conjugated secondary antibodies for 1 h at room temperature. For hemagglutinin (HA)-tagged protein detection, the membrane was incubated with HRP-conjugated antibodies. After washing with PBST three times, chemiluminescence was detected with the LAS4000 system (GE Healthcare, Chicago, IL, USA). Primary antibodies used for western blotting were as follows: anti-HA-peroxidase was purchased from Roche (# 12013819001, RRID: AB 390917); anti-FLAG M2 antibody was purchased from Sigma (# F1804-1MG, RRID: AB 262044); anti-green fluorescent protein (GFP) was purchased from Santa Cruz Biotechnology (#sc9996); anti-eEF2 (Lab stock); anti-eEF3 (Lab stock).

### Sucrose density gradient centrifugation

Yeast cells were grown exponentially at 30°C. Cells were harvested by centrifugation and the cell pellet was frozen and ground in liquid nitrogen using a mortar. The cell powder was resuspended with lysis buffer [20  mM HEPES-KOH pH 7.4, 100  mM potassium acetate, 2  mM magnesium acetate, 0.5 mM DTT, 1 mM PMSF, 1 tablet/10 ml complete mini EDTA-free (#11836170001, Roche)] to prepare the crude extracts. Sucrose gradients (10–50% sucrose in 10 mM Tris-acetate pH 7.4, 70  mM ammonium acetate and 4  mM magnesium acetate) were prepared in 14  ×  95  mm polyallomer tubes (Beckman Coulter) using a Gradient Master (BioComp). Crude extracts (the equivalent of 50 *A*_260_ units) were layered on top of the sucrose gradients and centrifuged at 28 500 *g* in a P40ST rotor (Hitachi Koki, Japan) for 1.5  at 4°C. The gradients were then fractionated with a BioComp Piston Gradient Fractionator. The polysome profiles were generated by continuous absorbance measurement at 254  nm using a single path UV-1 optical unit (ATTO Biomini UV-monitor) connected to a chart recorder (ATTO digital mini-recorder). For the western blots, 360 μl of each fraction was mixed with 40 μl of 100% TCA and incubated for 15 min at 4°C. After centrifugation (14 000 rpm, 15 min, 4°C), the supernatant was removed, and the pellet was washed with acetone and dissolved in 40 μl of SDS sample buffer (125 mM Tris–HCl pH 6.8, 4% SDS, 20% glycerol, 100 mM DTT, 0.01% bromophenol blue). The sample was heated at 100°C for 10 min and the resulting solution was separated by SDS–PAGE.

### Primer extension

Isolated total RNA was further treated with an equal volume of chloroform/isoamyl alcohol (24:1) and the water layer was collected into a new 1.5 ml RNase-free tube, followed by ethanol precipitation. A 60 μg aliquot of total RNA was subjected to reverse transcription by using SuperScript IV Reverse Transcriptase (Invitrogen Cat# 18090010) with 5′-IRDye700-labeled primer (LI-COR) complementary to the *HIS3* nucleotide sequence (5′-GCGATTGTGTGGCCTGTTCTGCTACTGCTTCTGCCTCTTTTTCTGGGAAGATCGAGTGCTCTATCGCTAGGGGACCACCC-3′) and the *FLAG* nucleotide sequence (5′-CTTGTCATCGTCGTCCTTGTAGTC-3′). An equal amount of phenol/chloroform/isoamyl alcohol (25:24:1) was added to the reaction and it was mixed by vortex. The water layer was collected after centrifugation, followed by cDNA precipitation with two-fifths the amount of 5 M NH_4_OAc, an equal volume of isopropanol and 1 μl of glycogen. The cDNA pellet obtained was dissolved in 8 μl of Stop Solution (95% formamide, 20 mM EDTA pH 8.0, 0.01% bromophenol blue). The cDNA sample was linearized at 70°C for 2 min followed by placing on ice for 5 min, and it was resolved on a 5% polyacrylamide–TBE–urea sequencing gel by electrophoresis at 1000 V for 150 min. Fluorescence of IRDye700 was detected by FLA-9000 (Fujifilm). The size of the reverse transcription product was determined by comparison with a sequencing ladder of corresponding reporter plasmid DNA prepared by using the same primer and the Thermo Sequenase Cycle Sequencing Kit (USB Cat# 78500 1 KT).

### Quantification and statistical analysis

All blot experiments were repeated at least twice, and a representative result is shown.

## RESULTS

### The N-terminal region of Cue2 is required for NGD that takes place in the colliding ribosome of the rare codons staller

**Figure 1.**
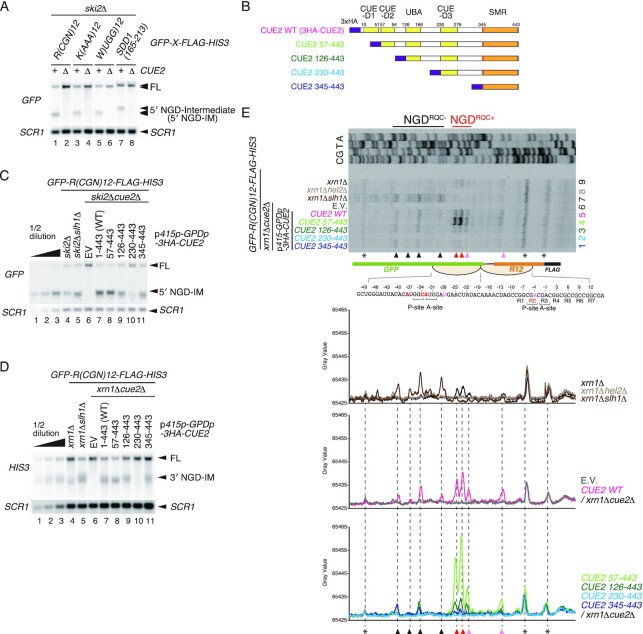
The N-terminal region of Cue2 is required for NGD^RQC+^, the subunit dissociation-dependent cleavage at the colliding ribosome. (**A**) Cue2 is generally required for NGD induced by various stalling sequences. Northern blot showing the 5′ NGD-IM derived from the *GFP-X-FLAG-HIS3* reporters containing the indicated stalling sequences, R(CGN)_12_, K(AAA)_12_, W(UGG)_12_ and SDD1(165–213) in *ski2*Δ or *ski2*Δ*cue2*Δ cells. 5′ NGD-IMs were detected with a DIG-labeled *GFP* probe. (**B**) Schematic drawing of the truncated Cue2 mutant proteins. (**C** and **D**) Northern blot analysis of NGD cleavage sites in Cue2 deletion mutants. The full-length *GFP-R(CGN)_12_-FLAG-HIS3* mRNA and 5′ NGD-IMs or 3′ NGD-IMs were detected in the indicated mutant cells by northern blotting with DIG-labeled probes (FL = full-length, EV = empty vector). 5′ NGD-IMs were detected by the DIG-labeled *GFP* probe and 3′ NGD-IMs were detected by the DIG-labeled *HIS3* probe. *SCR1* was used as a loading control. Note the downstream shift of NGD cleavage sites in lanes 9 and 11 (C), and the upstream shift of NGD cleavage sites in lanes 9 and 11 (D). (**E**) Top panel: primer extension mapping of 5′ ends of 3′ NGD-IMs in Cue2 wild type (WT) or the indicated Cue2 deletion mutant cells at nucleotide resolution. The primer extension samples were analyzed using 5% TBE–urea–PAGE and detected by fluorescence. Non-specific reverse transcription products are indicated by asterisks. The red arrowheads indicate the Cue2-mediated cleavage sites in the colliding ribosome in the presence of Slh1, and the black arrowheads indicate Cue2-mediated cleavage sites upstream of the colliding ribosome in the absence of the subunit dissociation by Slh1. Bottom panels: the pixel intensity was measured by the plot profile tool of ImageJ.

The Cue2 endonuclease has been identified as a crucial endonuclease for NGD in yeast (21). We confirmed the essential role of Cue2 in NGD induced by rare codons *R(CGN)_12_*, polytryptophan sequence *W(UGG)_12_* and the endogenous *poly(A)* and *SDD1* stalling sequences [*K(AAA)_12_* and *SDD1_165–213_*] (8,12,32) (Figure 1A). The 5′ NGD–mRNA intermediate (5′ NGD-IM) represents the primary product, which is rapidly degraded by the cytoplasmic exosome. Therefore, the 5′ NGD-IMs derived from various arrest-inducing sequences can be detected in mutants lacking the exosomal cofactor Ski2 (*ski2*Δ) (13,33). The 5′ NGD-IMs were detected in *ski2*Δ mutant cells, but no 5′ NGD-IMs were detected in *cue2*Δ*ski2*Δ mutant cells (Figure 1A), indicating a general role for Cue2 in NGD. Cue2 comprises three CUE domains and one UBA domain (Figure 1B), and these potential ubiquitin-binding domains may be responsible for the recognition of ubiquitinated ribosomes that are substrates for NGD. To investigate the roles of these domains in NGD, we constructed several deletion mutants of *CUE2*, expressed under the control of the *GPD1* promoter (Figure 1B). After checking the expression levels of mutant Cue2 proteins (Supplementary Figure S1A), we monitored a 5′ NGD-IM product derived from the *GFP-R(CGN)_12_-FLAG-HIS3* reporter mRNA [*R(CGN)_12_*]. Interestingly, we found two mutants, Cue2(126–443) and Cue2(345–443), which produced shorter 5′ NGD-IMs compared with the wild-type Cue2, indicating that mRNA cleavage sites shifted upstream (Figure 1C, lanes 9 and 11). Consistently, the corresponding 3′ NGD-IMs, which are degraded by the 5′–3′ exonuclease Xrn1, were longer, corroborating the upstream cleavage sites shift (Figure 1D, lanes 9 and 11). We also found Cue2(230–443) was defective in NGD (Figure 1C, lane 10; Figure 1D, lane 10), suggesting the inhibitory function of CUE-D3 to the cleavage by the SMR domain.

To obtain a more detailed insight into which parts of Cue2 are involved in the determination of cleavage sites in NGD pathways, we determined the precise endonucleolytic mRNA cleavage sites by primer extension. The 5′ ends of 3′-NGD-IMs derived from the *R(CGN)_12_* reporter mRNA in the *xrn1*Δ background were determined with a fluorescence-labeled primer that hybridized to the region of the HIS3-encoding mRNA sequence (Figure 1E). The ribosome is stalled mainly at positions R2(CGA) and R3(CGA) of the *R(CGN)_12_* sequence in P- and A-sites, respectively, and subjected to RQC (14). The mapping of the cleavage sites revealed that the cleavages take place within the collision site when Slh1-dependent subunit dissociation is intact as shown in *xrn1*Δ mutant cells (pink and red arrowheads, in Figure 1E, lane 9), and referred to as NGD^RQC+^. A minor cleavage site was in the P-site of the stalled ribosome (pink arrowhead, in Figure 1E, lane 9), and the main cleavage sites were in the mRNA region covered by the colliding ribosome (red arrowheads, in Figure 1E, lane 9). These cleavages depend on Hel2-mediated ubiquitination on uS10 at Lys6 and Lys8 residues and on Slh1 activity, indicating that the cleavage is induced upon subunit dissociation (13). In the absence of the Slh1 subunit dissociation activity, RQC is defective and the endonucleolytic mRNA cleavages take place only upstream of the collided ribosomes (NGD^RQC−^) as observed in the *xrn1*Δ*slh1*Δ mutant cells (black arrowheads, in Figure 1E, lane 7).

The mapping of the endonucleolytic mRNA cleavage sites induced by Cue wild type (Cue2-WT) (Figure 1E, lane 5) is almost the same as that in *xrn1*Δ mutant cells (Figure 1E, lane 9), but with higher intensities of the cleavage products. The band intensities of 5′ NGD-IM derived from the *R(CGN)_12_* reporter are consistent with this observation (Figure 1E, lower panels), indicating that cleavage efficiency correlates with the CUE2 expression level, and that NGD is a quantitative phenomenon in cells. Therefore, we investigated the relationship between Cue2 expression and cleavage efficiency, and found that the levels of 3′ NGD-IM derived from *R(CGN)_12_* indeed correlate with the expression levels of Cue2 (Supplementary Figure S1B, C). Unexpectedly, an endogenous *CUE2* promoter is not sufficient to complement the defects in *cue2*Δ mutant cells (Supplementary Figure S1B, lane 11; S1C, lane 8; and S1D, lane 2). The Cue2 expressed from the *GPD1* promoter, however, efficiently increased NGD (Supplementary Figure S1B, lanes 5 and 8), and the 5′ mapping by the primer extension revealed that both NGD^RQC+^ and NGD^RQC−^ were increased but their respective intensities are in proportion to the endogenous levels (Supplementary Figure S1C, lanes 5 and 8; and S1D, lanes 5 and 8). Therefore, we investigated the roles of ubiquitin-binding domains of Cue2 in NGD with CUE2 under the control of the *GPD1* promoter. In Cue2(57–443) mutant cells, the cleavage at the sites mapped within the collided ribosomes (NGD^RQC+^) was significantly increased but the upstream cleavage in the vicinity of the collided ribosome (NGD^RQC−^) was decreased (Figure 1E, lane 4). This suggests that CUE-D1 is involved in the upstream cleavage in the vicinity of the collided ribosome but dispensable for the cleavage at the sites mapped within the collided ribosomes. The cleavage at the sites mapped within the collided ribosomes (NGD^RQC+^) was significantly decreased in Cue2(126–443) mutant cells (Figure 1E, lane 3), suggesting that CUE-D2 or surrounding regions are involved in the NGD^RQC+^ cleavage. Consistent with the disappearance of 5′ NGD-IM and 3′ NGD-IM, no bands of the 3′ NGD-IM derived from the *R(CGN)_12_* reporter were detected in Cue2(230–443) mutant cells (Figure 1E, lane 2). The cleavage upstream of the collided ribosome (NGD^RQC−^) was detected in Cue2(345–443) mutant cells (Figure 1E, lane 1), suggesting an inhibitory function of CUE-D3 on cleavage in the vicinity of the stalled ribosome by the SMR domain. Taken together, the analysis of NGD in *CUE2* deletion mutants strongly suggests a function for CUE-D1 and CUE-D2 in the determination of the cleavage sites corresponding to NGD^RQC+^ or NGD^RQC−^.

### CUE-D1 and CUE-D2 domains are required for NGD^RQC−^ but dispensable for NGD^RQC+^

**Figure 2.**
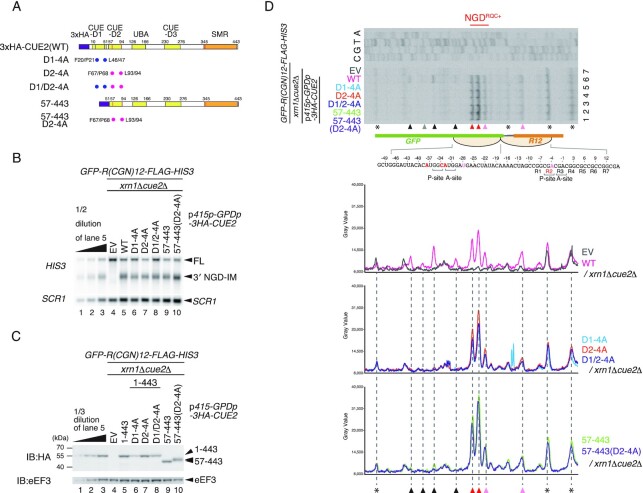
CUE-D1 and CUE-D2 domains are required for NGD^RQC−^ but dispensable for NGD^RQC+^. (**A**) Schematic drawing of the N-terminal HA-tagged Cue2 mutant proteins. The blue dots indicate alanine substitutions of D1-4A, and the pink dots indicate alanine substitutions of D2-4A. (**B**) Northern blot analysis of NGD cleavage sites in the Cue2 mutants shown in (A). The full-length *GFP-R(CGN)_12_-FLAG-HIS3* mRNA and 3′ NGD-IMs were detected in the indicated mutant cells by northern blotting with the DIG-labeled *HIS3* probe. *SCR1* was used as a loading control. FL = full-length. (**C**) Western blot analysis to check the expression levels of HA-tagged Cue2 mutant proteins as schematically displayed in (A) using an anti-HA antibody. (**D**) Top panel: primer extension mapping of 5′ ends of 3′ NGD-IMs in Cue2-WT or the indicated Cue2 mutant cells at nucleotide resolution. The primer extension samples were analyzed using 5% TBE–urea–PAGE and detected by fluorescence. Non-specific reverse transcription products are indicated by asterisks. Bottom panels: the pixel intensity was measured by the plot profile tool of ImageJ.

To obtain a more detailed insight into the roles of CUE-D1 and CUE-D2 in the determination of the cleavage sites, we introduced mutations in these domains that disrupt their ubiquitin binding function. The conserved residues required for ubiquitin binding in CUE-D1 and CUE-D2 domains were substituted with alanine residues (Figure 2A). In the CUE-D1 domain, phenylalanine 20 (F20), proline 21 (P21), leucine 46 (L46) and leucine 47 (L47) were replaced by alanine residues (D1-4A, blue dots in Figure 2A). In the CUE-D2 domain, phenylalanine 67 (F67), proline 68 (P68), leucine 93 (L93) and leucine 94 (L94) residues were substituted with alanine residues (D2-4A, pink dots in Figure 2A). We created Cue2 mutants containing D1-4A or D2-4A alone and in combination, and monitored the 3′ NGD-IM product derived from *R(CGN)_12_* reporter mRNA. No band up-shift or down-shift was observed for the 3′ NGD-IM product (Figure 2B, lanes 5–10), suggesting that the cleavage in these mutants occurred mainly within the collided ribosomes (NGD^RQC+^). We also checked the expression levels of the mutant Cue2 proteins which were comparable (Figure 2C), although the expression level of Cue2-D1-4A was somewhat lower than in wild-type Cue2(1–443) or other mutant variants (Figure 2C, lanes 5 and 6). To dissect the cleavage sites of the Cue2 mutants, we determined the 5′ end of 3′ NGD-IM derived from the *R(CGN)_12_* reporter mRNA in the *xrn1*Δ background by primer extension experiments. In CUE2-WT, the cleavage mainly takes place within the collided ribosomes (NGD^RQC+^) but there are minor cleavages upstream of the colliding ribosome (NGD^RQC−^) (Figure 2D, lane 6). In all Cue2 mutants, minor cleavages upstream of the collided ribosome were eliminated, while the cleavages within the collided ribosomes were increased (Figure 2D, lanes 1–5; lower panels). These results suggest that CUE-D1 and CUE-D2 domains are required for NGD^RQC−^ upstream of the collided ribosomes but are dispensable for NGD^RQC+^ within the collided ribosomes.

### The Trp122 residue of Cue2 is specifically required for NGD^RQC+^ in the colliding ribosome

**Figure 3.**
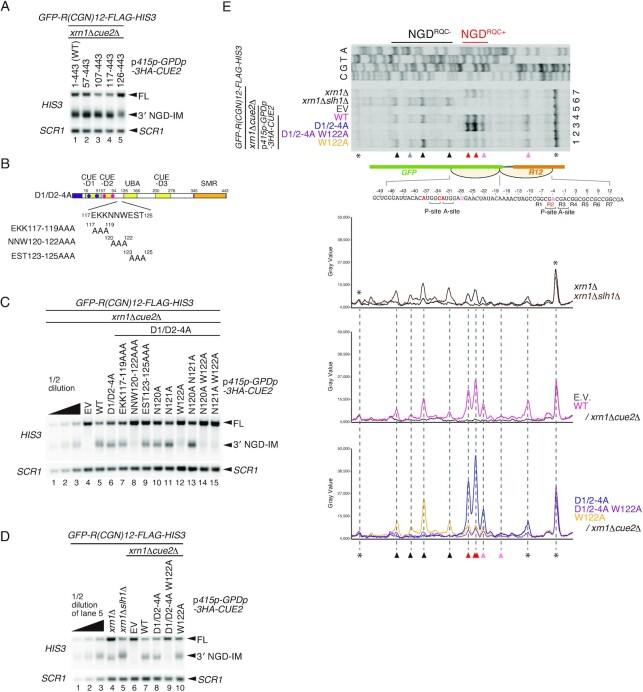
The Trp122 residue of Cue2 is specifically required for NGD^RQC+^ in the colliding ribosome. (**A**) Northern blot analysis of NGD cleavage sites in the Cue2 deletion mutants. The full-length *GFP-R(CGN)_12_-FLAG-HIS3* mRNA and 3′ NGD-IMs were detected in the indicated mutant cells by northern blotting with DIG-labeled probes. 3′ NGD-IMs were detected by the DIG-labeled *HIS3* probe. *SCR1* was used as a loading control. FL = full-length. (**B**) Schematic drawing of the N-terminal HA-tagged Cue2 mutant proteins containing mutations in residues 117–125. The blue dots indicate alanine substitutions of D1-4A, and the pink dots indicate alanine substitutions of D2-4A. (**C**) Northern blot analysis of NGD cleavage sites in the Cue2 mutants shown in (A), and single or double alanine substitutions in residues 120–122. The full-length *GFP-R(CGN)_12_-FLAG-HIS3* mRNA and 3′ NGD-IMs were detected in the indicated mutant cells by northern blotting with DIG-labeled probes. (**D**) Northern blot analysis of NGD cleavage sites in the Cue2 mutants containing the mutations D1/D2-4A and/or W122A. The full-length *GFP-R(CGN)_12_-FLAG-HIS3* mRNA and 3′ NGD-IMs were detected in the indicated mutant cells by northern blotting with DIG-labeled probes. (**E**) Top panel: primer extension mapping of 5′ ends of 3′ NGD-IMs in Cue2-WT or the indicated Cue2 mutant cells at nucleotide resolution. The primer extension samples were analyzed using 5% TBE–urea–PAGE and detected by fluorescence. Non-specific reverse transcription products are indicated by asterisks. Bottom panels: the pixel intensity was measured by the plot profile tool of ImageJ.

Although the CUE-D1 and CUE-D2 domains are dispensable for NGD^RQC+^, the cleavage inside the collided ribosomes was significantly decreased in Cue2(126–443) mutant cells in comparison with Cue2(57–443) mutant cells (Figure 1E, lanes 3 and 4). We suspected that CUE2-D2 or surrounding regions are involved in cleavage at the sites mapped within the collided ribosomes. To test this, we constructed Cue2 deletion mutants and monitored 3′ NGD-IM products derived from *R(CGN)_12_* reporter mRNA after checking the expression levels of mutant Cue2 proteins (Supplementary Figure S2). We observed no up- or down-shift of the 3′ NGD-IM product band (Figure 3A), but its intensity was decreased in Cue2(126–443) mutant cells (Figure 3A, lane 5). This suggests that the 117–125 amino acid region but not the CUE2-D2 domain, 57–94 amino acid region, is involved in the cleavage in the collided ribosomes. To further investigate this, we introduced alanine substitutions into the 117–125 amino acid region (Figure 3B) in combination with D1/D2-4A mutations and determined the levels of 3′ NGD-IM products. No 3′ NGD-IM product was detected in Cue2 mutants harboring a substitution of the ^120^NNW^122^ sequence with alanine residues in combination with the D1/D2-4A mutation (Figure 3C, lane 8). This suggests that this region is crucial for NGD^RQC+^ within the disome. Therefore, we further tested if single-point alanine substitution mutants in the 120–122 region would disrupt the NGD in D1/D2-4A *CUE2* mutant cells. Notably, 3′ NGD-IM product was significantly reduced in the W122A substitution Cue2 mutant (Figure 3C, lane 12) while no such effect took place in the N120A or the N121A substitution mutants (Figure 3C, lanes 10 and 11). Since only the NGD^RQC+^ is possible in D1/D2-4A *CUE2* mutant cells (Figure 2D, lane 3), this suggests that this activity critically depends on the W122 residue. As expected, the W122A mutation on its own did not abrogate the NGD as the NGD^RQC−^ functions in cells with unmutated CUE-D1 and CUE-D2 domains (Figure 3D, lane 10). This confirms the importance of the W122 residue specifically for the NGD^RQC+^. To determine the cleavage site preferences of these Cue2 mutants, we determined the 5′ ends of 3′ NGD-IM by primer extension. The bands corresponding to NGD^RQC+^ are evident in the D1/D2-4A *CUE2* mutant cells but absent in the D1/D2-4A W122A *CUE2* mutant cells (Figure 3E, lanes 2 and 3). Consistently, NGD^RQC+^ cleavages within the collided ribosomes were dramatically decreased in the W122A *CUE2* mutant cells without the D1/D2-4A mutation while NGD^RQC−^ cleavages upstream of it were enhanced (Figure 3E, lanes 1 and 4; lower panels). These results together show that the W122 residue of Cue2 is specifically and critically required for the NGD^RQC+^ endonucleolytic mRNA cleavage within the collided ribosomes.

### Conserved roles of Cue2 in two modes of NGD induced by the endogenous *SDD1* staller

The ribosome collision caused by strong translation stalling on rare codons result in two modes of NGD, and our data show that N-terminal CUE domains are required for NGD^RQC−^ while the W122 residue is specifically required for NGD^RQC+^. Since Cue2 is a general NGD factor as is Hel2 (Figure 1A), we asked whether this Cue2 mode of action is conserved in NGD induced by endogenous mRNA stallers.

**Figure 4.**
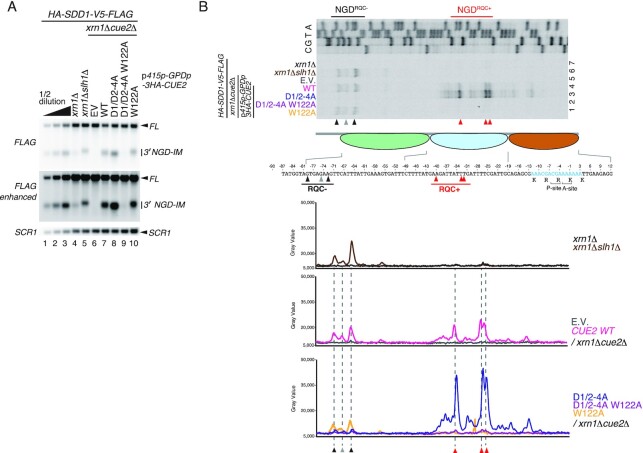
Conserved roles of CUE domains for two modes of NGD in the *SDD1* staller. (**A**) Northern blot analysis of NGD cleavage sites in the Cue2 mutants containing the mutations D1/D2-4A and/or W122A. The full-length *HA-SDD1-V5-FLAG* mRNA and 3′ NGD-IMs were detected in the indicated mutant cells by northern blotting with DIG-labeled probes. 3′ NGD-IMs were detected by the DIG-labeled *FLAG* probe. *SCR1* was used as a loading control. FL = full-length. Note the upstream shift of NGD cleavage sites in lanes 5 and 10. (**B**) Primer extension mapping of 5′ ends of 3′ NGD-IMs in Cue2-WT or the indicated Cue2 mutant cells at nucleotide resolution. The primer extension samples were analyzed using 5% TBE–urea–PAGE and detected by fluorescence. Non-specific reverse transcription products are indicated by asterisks. The red arrowheads indicate the Cue2-mediated cleavage sites in the second colliding ribosome in the presence of Slh1, and the black arrowheads indicate Cue2-mediated cleavage sites upstream of the third colliding ribosome in the absence of the subunit dissociation by Slh1. Bottom panels: the pixel intensity was measured by the plot profile tool of ImageJ.

Recent studies demonstrated that the endogenous *SDD1* stalling sequence efficiently induces di- but also tri-ribosome formation and RQC (12). Since Cue2 is required for NGD caused by the *SDD1* staller (Figure 1A, lanes 7 and 8), we examined the role of the ubiquitin binding activity of Cue2 in NGD^RQC−^ or NGD^RQC+^ in this case. The 3′ NGD-IM derived from the *HA-SDD1-V5-FLAG* reporter, which is degraded by the 5′–3′ exonuclease Xrn1, was detected in *xrn1*Δ mutant cells (Figure 4A, lanes 4 and 7). A significant increase and up-shift of the 3′ NGD-IM was observed in *slh1*Δ*xrn1*Δ mutant cells, suggesting cleavage upstream of the collided ribosomes (NGD^RQC−^) in the absence of Slh1 (Figure 4A, lane 5). To obtain a more detailed insight into NGD induced by *SDD1* mRNA and the role of Cue2 in the determination of cleavage sites, we resolved the precise endonucleolytic mRNA cleavage sites by primer extension assay (Figure 4B). The leading ribosome is stalled on the *SDD1* mRNA mainly at positions R(CGA) and K(AAA) in P- and A-sites, respectively, and subjected to RQC (12). The mapping of the cleavage sites revealed that the major cleavages in the *SDD1* staller in *xrn1*Δ mutant cells (red arrowheads, in Figure 4B, lane 4) are analogous to the NGD^RQC+^ cleavage sites in the rare codon-collided ribosomes (Figure 1E). In the absence of Slh1, the cleavages take place upstream of the collided ribosomes in *slh1*Δ*xrn1*Δ mutant cells (black arrowheads, in Figure 4B, lane 6) analogously to NGD^RQC−^ cleavages in the rare codons staller (Figure 3E). In D1/D2-4A CUE2 mutant cells, the NGD^RQC+^ cleavages within the collided ribosomes were increased and the cleavages upstream of collided ribosomes (NGD^RQC−^) were disrupted (Figure 4B, lane 3). The enhanced cleavages in the colliding ribosomes (NGD^RQC+^) in D1/D2-4A CUE2 mutant cells were eliminated by the W122A mutation (Figure 4B, lane 2). These results confirm that the function of Cue2 in NGD induced by the *SDD1* staller is consistent with that induced by the strong rare codon *R(CGN)_12_* reporter mRNA. Both the CUE-D1 and the CUE-D2 domains are required for NGD^RQC−^ upstream of the endogenous *SDD1* staller but dispensable for NGD^RQC+^, and the W122 residue is specifically required only for the NGD^RQC+^ cleavage within the collided ribosomes.

### Mbf1 is required for quality control by preventing +1 RFS induced by a rare codon staller

**Figure 5.**
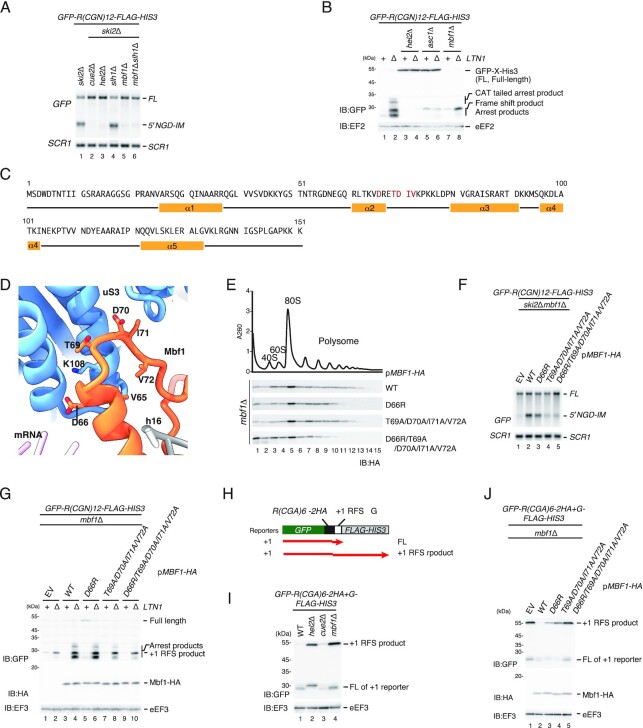
Mbf1 is indispensable for RQC and NGD induced by the rare codon stalling mRNA. (**A**) Mbf1 is required for NGD by a rare codons staller. The full-length *GFP-R(CGN)_12_-FLAG-HIS3* mRNA and 5′ NGD-IMs were detected in the indicated mutant cells by northern blotting with DIG-labeled *GFP* probes. *SCR1* was used as a loading control. FL = full-length. (**B**) Mbf1 is required for RQC by a rare codons staller. Western blot of the arrest products derived from *GFP-R(CGN)_12_-FLAG-HIS3* mRNA. The arrest and +1 RFS products derived from the *GFP-R(CGN)_12_-FLAG-HIS3* mRNA reporter in the indicated mutant cells were detected with western blotting using an anti-GFP antibody. (**C**) Schematic drawing of the Mbf1 sequence and secondary structure. (**D**) Molecular model of the Mbf1–uS3 interaction site near mRNA entry and helix 16 (PDB: 6ZVI). Residues of Mbf1 and uS3 important for the interaction are shown as sticks (D66, T69, D70, I71, V72 of Mbf1; K108 of uS3). (**E**) The ribosome association of Mbf1 mutants. Cell lysates derived from *mbf1* mutant cells expressing the indicated Mbf1 mutant protein were subjected to ultracentrifugation in sucrose density gradients. Gradient fractions were used for western blotting with anti-HA antibody. (**F**) Northern blot analysis of NGD cleavage sites in the indicated Mbf1 mutant cells. The full-length *GFP-R(CGN)_12_-FLAG-HIS3* mRNA and 5′ NGD-IMs were detected in the indicated mutant cells by northern blotting with DIG-labeled *GFP* probes. *SCR1* was used as a loading control. FL = full-length. (**G**) Western blot of the arrest product derived from *GFP-R(CGN)_12_-FLAG-HIS3* in the indicated *mbf1* mutants. The arrest products derived from the *GFP-R(CGN)_12_-FLAG-HIS3* reporter in the indicated mutant cells was detected with western blotting using an anti-GFP antibody. Mbf1-HA proteins were detected with western blotting using an anti-HA antibody. (**H**) The schematic presentation of the 0 RFS and +1 RFS reporter. The black lines indicate the products derived from *GFP-R(CGA)_6_–2HA-FLAG-HIS3* (0 RFS) and red lines indicate the products derived from *GFP-R(CGA)_6_–2HA-+G-FLAG-HIS3* (+1 RFS). (**I**) Mbf1 represses +1 RFS by a rare codons staller. The products derived from *GFP-R(CGA)_6_–2HA-FLAG-HIS3* (lanes 1–4) and *GFP-R(CGA)_6_–2HA-+G-FLAG-HIS3* (lanes 5–8) in wild-type and the indicated mutant cells were detected by western blotting using an anti-GFP antibody. (**J**) The efficiency of +1 RFS by a rare codons staller in the *mbf1* mutant cells. The products derived from *GFP-R(CGA)_6_–2HA-FLAG-HIS3* (lanes 1–4) and *GFP-R(CGA)_6_–2HA-+G-FLAG-HIS3* (lanes 5–8) in wild-type and the indicated *mbf1* mutant cells were detected by western blotting using an anti-GFP antibody. Mbf1-HA proteins were detected with western blotting using an anti-HA antibody.

Mbf1 is associated with the colliding ribosome and acts with ribosomal proteins Rps3/uS3 and Asc1/RACK1 to prevent frameshifting at inhibitory CGA–CGA codon pairs in yeast (23,24). However, there is no direct evidence to show the essential roles of Mbf1 in RQC and NGD. Moreover, there is no comparison between the efficiency of +1 RFS and that of NGD/RQC induced by various stallers. To further investigate the role of Mbf1 in RQC and NGD induced by the various stallers, we first detected the 5′ NGD-IM product derived from *R(CGN)_12_* reporter mRNA in *ski2*Δ and the shorter NGD^RQC−^ 5′ NGD-IM in *slh1*Δ*ski2*Δ mutant cells (Figure 5A, lanes 1 and 4) as previously reported (13). However, no 5′ NGD-IM was detected in *cue2*Δ*ski2*Δ, *mbf1*Δ*ski2*Δ or *mbf1*Δ*slh1*Δ*ski2*Δ mutant cells (Figure 5A, lanes 2, 5 and 6). This indicates that Mbf1 is required for both the NGD^RQC+^ and NGD^RQC–^ induced by the *R(CGN)_12_* rare codon staller. We also investigated the function of Mbf1 in RQC induced by the rare codon-collided ribosomes. The arrest product derived from *R(CGN)_12_* reporter mRNA detected in the *ltn1*Δ background (Figure 5B, lane 2) was not detectable in *hel2*Δ*ltn1*Δ, *asc1*Δ*ltn1*Δ and *mbf1*Δ*ltn1*Δ mutant cells (Figure 5B, lanes 4, 6 and 8), indicating that Mbf1 is indispensable also for RQC induced by the *R(CGN)_12_* rare codon staller.

To further investigate the role of the Mbf1–ribosome interaction in quality control pathways, we introduced mutations in residues of Mbf1 that contribute to the interaction with uS3 (Figure 5C, D). The association of WT and mutant Mbf1 with ribosomes was tested by western blotting using the fractions from ultracentrifugation in sucrose density gradients (Figure 5E). We observed that the ribosome association of Mbf1 was moderately diminished in Mbf1–T69A/D70A/I71A/V72A mutant cells and significantly in Mbf1–D66R/T69A/D70A/I71A/V72A mutant cells (Figure 5E). We then investigated whether the Mbf1–ribosome interaction is required for quality control pathways induced by the rare codons staller. The 5′ NGD-IM product derived from *R(CGN)_12_* reporter mRNA was detected in the Mbf1 wild-type *ski2*Δ background (Figure 5F, lane 2), but was drastically decreased in Mbf1–D66R/T69A/D70A/I71A/V72A mutant cells (Figure 5F, lane 5), confirming the important role of Mbf1 in NGD induced by the rare codons staller. In the *ltn1*Δ background, the arrest product derived from the *R(CGN)_12_* reporter mRNA was detected for wild-type Mbf1 (Figure 5G, lane 4) but was eliminated in the Mbf1–D66R/T69A/D70A/I71A/V72A quintuple mutant cells (Figure 5G, lane 10). These results indicate that the Mbf1–uS3 interaction is indispensable for RQC and NGD induced by the rare codons staller.

It has been reported that ribosome binding of Mbf1 is crucial for reading frame maintenance during ribosome stalling by rare codons stallers (23,24). Therefore, we tested the role of Mbf1 together with Cue2 and Hel2 using the *GFP-R(CGA)6–2HA-+G-FLAG-HIS3* reporter (Figure 5H). In agreement with previously reported findings (23,24,29), the +1 RFS product derived from the *GFP-R(CGA)_6_–2HA-+G-FLAG-HIS3* reporter was detected in *mbf1*Δ mutant cells with larger products than the arrest products (Figure 5I, lane 4). The level of +1 RFS product was increased in *hel2*Δ (Figure 5I, lane 2), suggesting that the Hel2-initiated RQC and NGD represses +1 RFS. The level of +1 RFS product was not increased in *cue2*Δ cells (Figure 5I, lane 3), suggesting that a lack of Cue2-mediated endonucleolytic cleavage during the ribosome stalling does not affect +1 RFS. We then determined the efficiency of +1 RFS by the rare codons staller in the *mbf1* substitution mutants (Figure 5J). The level of +1 RFS products derived from the *GFP-R(CGA)_6_–2HA-+G-FLAG-HIS3* reporter was drastically increased in the *mbf1* mutant cells (Figure 5J, lanes 3–5). The +1 RFS efficiency estimated with the level of the +1 RFS product (Figure 5J) is consistent with that of the defects in NGD (Figure 5F) and RQC (Figure 5G), strongly suggesting that the defects in RQC and NGD may be due to the increase of the +1 RFS.

### Mbf1 contributes to quality control pathways induced by ribosome stalling but its roles differ among the stalling sequences

**Figure 6.**
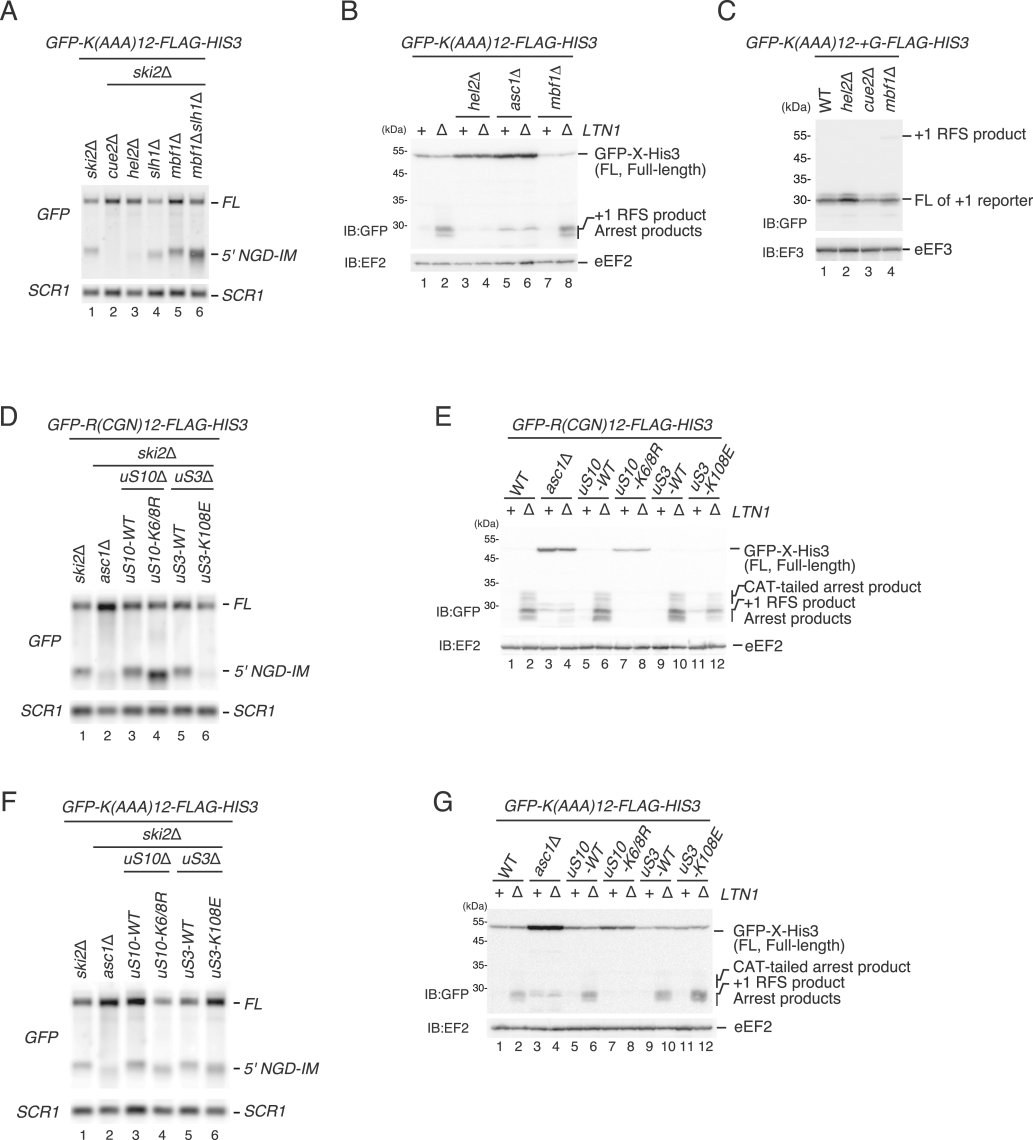
Mbf1 and Mbf1–uS3 interaction are dispensable for RQC and NGD induced by the poly(A) staller. (**A**) Mbf1 is dispensable for NGD induced by the poly(A) staller. 5′ NGD-IMs derived from the *GFP-K(AAA)_12_-FLAG-HIS3* reporter were detected in the indicated mutant cells by northern blotting with a DIG-labeled *GFP or SCR1* probe. The 5′ NGD-IM is shortened in *uS10-K6/8R* mutant cells, indicating that the endonucleolytic mRNA cleavage site is shifted upstream. (**B**) Mbf1 is dispensable for RQC induced by the poly(A) staller. The arrest products derived from the *GFP-K(AAA)_12_-FLAG-HIS3* reporter were detected in the W303*ski2*Δ, *asc1*Δ*ski2*Δ, *uS10-K6/8R ski2*Δ, *uS3-WTski2*Δ or *uS3-K108Eski2*Δ mutant cells by western blotting using an anti-GFP antibody. (**C**) The +1 RFS product by the poly(A) staller was not detected in *mbf1* mutant cells. The products derived from *GFP-K(AAA)_12_-+G-FLAG-HIS3* in wild-type and the indicated mutant cells were detected by western blotting using an anti-GFP antibody. (**D**) Mbf1–uS3 interaction is required for NGD by the rare codon staller. 5′-NGD-IMs derived from the *GFP-R(CGN)_12_-FLAG-HIS3* reporter were detected in in the W303*hel2*Δ, *uS3-WT*, *asc1*Δ, *uS10-K6/8R uS3-K108E* mutant cells cells by northern blotting with a DIG-labeled *GFP* or *SCR1* probe. The 5′-NGD-IMs are shortened in *uS10-K6/8R* mutant cells, indicating that the endonucleolytic mRNA cleavage site is shifted upstream. (**E**) Mbf1–uS3 interaction is required for RQC by the rare codon staller. The arrest products in the *ltn1*Δ background derived from the *GFP-R(CGN)_12_-FLAG-HIS3* reporter were detected in the indicated mutant cells by western blotting with an anti-GFP antibody. (**F**) The K108 of uS3 is dispensable for NGD induced by the poly(A) staller. 5′ NGD-IMs derived from the *GFP-K(AAA)_12_-FLAG-HIS3* reporter were detected in the indicated mutant cells by northern blotting with a DIG-labeled *GFP* or *SCR1* probe. (**G**) The K108 of uS3 is dispensable for RQC induced by the poly(A) staller. The arrest products in the *ltn1*Δ background derived from the *GFP-K(AAA)_12_-FLAG-HIS3* were detected in the W303*hel2*Δ, *uS3-WT*, *asc1*Δ and *uS10-K6/8R uS3-K108E* mutant cells using an anti-GFP antibody.

To further dissect the role of Mbf1 in NGD, we used a poly(A) staller mRNA. Collided ribosomes stalled on a poly(A) stretch were reported to largely adopt a POST–POST collision conformation (34), while Mbf1 was suggested to specifically interact with the second colliding ribosome in a hybrid state (25,26). Therefore, the specific ribosome collision conformation induced by poly(A) stalling could potentially prevent Mbf1 interaction with the colliding ribosome(s). Interestingly, we found that NGD^RQC+^ induced by the poly(A)-stalled and collided ribosomes is not reduced in the *mbf1*Δ mutant cells (Figure 6A, lane 1, *ski2*Δ and lane 5, *mbf1*Δ*ski2*Δ), and the arrest product was detected in the *ltn1*Δ*mbf1*Δ mutant (Figure 6B, lane 2, *ltn1*Δ and lane 8, *mbf1*Δ*ltn1*Δ). We then examined the role of Mbf1 in the maintenance of the reading frame during ribosome stalling on a poly(A) tract. The +1 RFS product derived from the *GFP-K(AAA)_12_-+G-FLAG-HIS3* reporter was not increased in *hel2*Δ, *cue2*Δ or *mbf1*Δ mutant cells (Figure 6C, lanes 2–4). In *hel2*Δ mutant cells, ribosome stalling by the poly(A) sequence was reduced (Figure 6B, lanes 3 and 4); however, +1 RFS product was not detected (Figure 6C, lane 2). Taken together, Mbf1 contributes to quality control pathways induced by ribosome stalling but its roles are different depending on the stalling sequences.

To investigate the role of uS3 as the ribosomal binding partner of Mbf1, we examined the defect in RQC and NGD in the substitution mutant of uS3 at the K108 residue, a crucial site for the interaction with Mbf1 and reading frame maintenance (35). Both RQC and NGD induced by the rare codon-collided ribosomes were eliminated in the single point *uS3-K108E* mutant cells (Figure 6D, E). The *uS3-K108E* mutation disrupted the cleavage of the rare codon staller mRNA as no 5′ NGD-IM derived from *GFP-R(CGN)_12_-FLAG-HIS3* mRNA was detected in *ski2*Δ*uS3-K108E* cells (Figure 6D, lane 6), suggesting that the interaction between uS3 and Mbf1 may be required for NGD induced by the rare codon staller. The arrest product derived from the *GFP-R(CGN)_12_-FLAG-HIS3* reporter was not detected but the truncated +1 RFS products were detected in *ltn1*Δ*uS3-K108E* cells (Figure 6E, lane 12). Together, these results demonstrate that both RQC and NGD induced by the rare codon staller were defective in the *uS3-K108E* mutant cells. On the other hand, for the poly(A) staller, we were able to detect the 5′ NGD-IM derived from *GFP-K(AAA)_12_-FLAG-HIS3* mRNA (Figure 6F, lane 6), and the arrest product derived from *GFP-R(AAA)_12_-FLAG-HIS3* reporter (Figure 6G, lane 12), suggesting that the interaction of uS3 with Mbf1 is required to stabilize the rare codon-stalled collided ribosomes but not the poly(A)-stalled collided ribosomes.

### Mbf1 is required for the NGD^RQC+^ cleavage at the colliding ribosome of the *SDD1* staller

**Figure 7.**
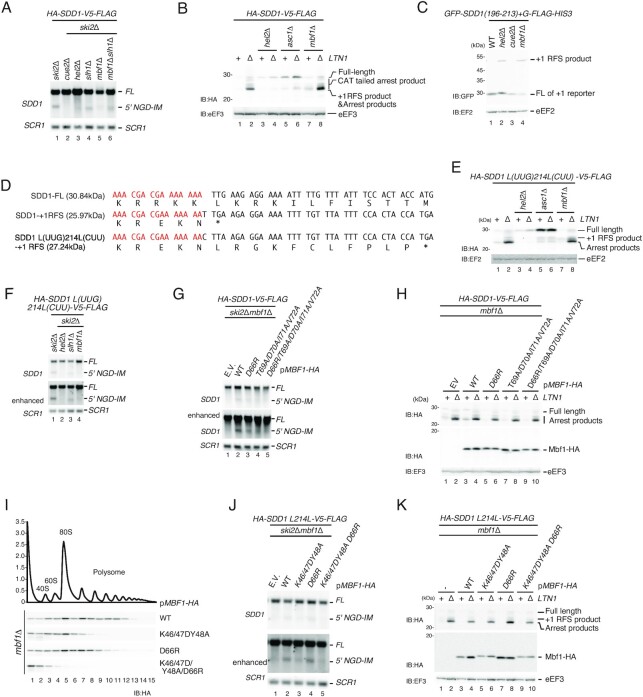
Mbf1 is required for the cleavage at the second colliding ribosome of the *SDD1* staller. (**A**) Northern blot analysis of NGD cleavage sites in the indicated mutant cells. The full-length *HA-SDD1-V5-FLAG* mRNA and 5′ NGD-IM were detected in the indicated mutant cells by northern blotting with DIG-labeled *SDD1* probes. *SCR1* was used as a loading control. FL = full-length. (**B**) RQC induced by the *SDD1* staller is intact in the absence of Mbf1. The arrest products derived from the *HA-SDD1-V5-FLAG* in *ltn1*Δ cells were detected with an anti-HA antibody. (**C**) Mbf1 represses +1 RFS by the *SDD1* staller. The products derived from *GFP-SDD1(196–213)+G-FLAG-HIS3* in wild-type or the indicated mutant cells were detected by western blotting using an anti-GFP antibody. (**D**) Schematic drawing of the putative *SDD1* translation products. +1 RFS indicates a product of the putative +1 frameshifting at the stalling sequence (25.97 kDa). A putative frameshift product derived from *SDD1-L(UUG)214L(CUU)* is 27.24 kDa and is distinguishable from the arrest product produced by RQC (∼24 kDa). (**E**) Western blot of the arrest product derived from *SDD1-L(UUG)214L(CUU)* suggests that RQC induced by the *SDD1* staller is intact in the *mbf1* mutant cells. The arrest products derived from the *SDD1-L(UUG)214L(CUU)* reporters in the indicated mutant cells were detected with western blotting using an anti-HA antibody. (**F**) Northern blot analysis of NGD cleavage sites in the indicated mutant cells. The full-length *HA-SDD1-L(UUG)214L(CUU)* mRNA and 5′ NGD-IMs were detected in the indicated mutant cells by northern blotting with DIG-labeled *SDD1* probes. *SCR1* was used as a loading control. FL = full-length. (**G**) Northern blot analysis of NGD cleavage sites in the indicated *mbf1* mutant cells. The full-length *HA-SDD1-V5-FLAG* mRNA and 5′ NGD-IMs were detected in the indicated mutant cells by northern blotting with DIG-labeled *SDD1* probes. *SCR1* was used as a loading control. FL = full-length. (**H**) Western blot of the arrest product derived from *HA-SDD1-V5-FLAG*. The arrest products derived from the *HA-SDD1-V5-FLAG* reporter in the indicated mutant cells were detected with western blotting using an anti-HA antibody (top and bottom panels), and anti-eEF3 (bottom panel). (**I**) The ribosome association of Mbf1 mutants defective in mRNA interaction. Cell lysates derived from *mbf1* mutant cells expressing the indicated Mbf1 mutant protein were subjected to ultracentrifugation in sucrose density gradients. Gradient fractions were used for western blotting with anti-HA antibody (**J**) Northern blot analysis of NGD cleavage sites in the indicated *mbf1* mutant cells. The full-length *HA-SDD1-V5-FLAG* mRNA and 5′ NGD-IMs were detected in the indicated mutant cells by northern blotting with DIG-labeled *GFP* probes. *SCR1* was used as a loading control. FL = full-length. (**K**) Western blot of the arrest product derived from *HA-SDD1-V5-FLAG*. The arrest products derived from the *HA-SDD1-V5-FLAG* reporter in the indicated mutant cells were detected with western blotting using an anti-HA antibody (top and bottom panels) and anti-eEF3 (bottom panel).

We mapped the Cue2-mediated NGD^RQC+^ cleavages specifically in the area covered by the second colliding ribosome on the *SDD1* mRNA (Figure 4B). One possibility to explain such specificity is the role of a putative factor specifically associated with the second colliding ribosome which may contribute to recruiting Cue2 to the cleavage site. The Mbf1 structures demonstrate that Mbf1 associates with colliding ribosomes but not with the first stalled one (25,26). Therefore, we examined whether Mbf1 is involved in NGD induced by the *SDD1* staller by monitoring the 5′ NGD-IM product derived from *HA-SDD1-V5-FLAG* mRNA (Figure 7A). No signal of the 5′ NGD-IM was observed in *cue2*Δ*ski2*Δ and *hel2*Δ*ski2*Δ mutant cells (Figure 7A, lanes 2 and 3), indicating that Hel2 and Cue2 are required for NGD induced by the *SDD1* staller. We observed the downshift of 5′ NGD-IM derived from *HA-SDD1-V5-FLAG* mRNA in *ski2*Δ*slh1*Δ and *ski2*Δ*slh1*Δ*mbf1*Δ mutant cells (Figure 7A, lanes 4 and 6). As in previous experiments, the downshift of 5′ NGD-IM served as an indicator of NGD^RQC−^ (shorter 5′ NGD-IM product generated by upstream mRNA cleavage). These results suggest that the NGD^RQC–^ upstream cleavage in the *SDD1* staller mRNA takes place in the absence of the subunit dissociation, and Mbf1 is not involved in NGD^RQC−^ by the *SDD1* staller. On the other hand, we did not observe any 5′ NGD-IM band in the *mbf1*Δ*ski2*Δ mutant cells (Figure 7A, lane 5). Therefore, Mbf1 is required for the ribosome subunit dissociation-coupled cleavage in the collided ribosomes (NGD^RQC+^) but not for the upstream cleavage independent of the subunit dissociation (NGD^RQC−^). We also investigated the function of Mbf1 in RQC induced by the *SDD1* staller. The arrest product derived from *HA-SDD1-V5-FLAG* mRNA detected in the *ltn1*Δ background (Figure 7B, lane 2) was substantially reduced in *hel2*Δ*ltn1*Δ mutant cells and *asc1*Δ*ltn1*Δ mutant cells (Figure 7B, lanes 4 and 6). On the other hand, unreduced levels of the arrest product were detected in *mbf1*Δ*ltn1*Δ mutant cells (Figure 7B, lane 8), suggesting that Mbf1 is dispensable for RQC induced by the *SDD1* staller.

Since +1 RFS by rare codons was increased in *mbf1*Δ*ltn1*Δ mutant cells (Figure 5I) (23,24,29), we tested the +1 RFS induced by the *SDD1* staller using the *GFP-SDD1(196–213)-+G-FLAG-HIS3* reporter (Figure 7C). As expected, the +1 RFS product derived from the *GFP-SDD1(196–213)-+G-FLAG-HIS3* reporter was detected in *mbf1*Δ mutant cells (Figure 7C, lane 4). The level of +1 RFS product was also increased in *hel2*Δ cells (Figure 7C, lane 2), suggesting that the Hel2-initiated RQC and NGD represses +1 RFS. The level of +1 RFS product was not increased in *cue2*Δ cells (Figure 7C, lane 3), suggesting that a lack of cleavage during the ribosome stalling does not affect +1 RFS by the *SDD1* staller. We then examined the +1 RFS products derived from *HA-SDD1-FLAG*. The size of the arrest product (25.70 kDa) and the putative +1 RFS product (25.97 kDa) would be almost the same and it would be difficult to distinguish them by western blotting. To distinguish the arrest product from the putative +1 RFS product in *mbf1*Δ*ltn1*Δ mutant cells, we introduced the L(UUG)214 to L(CUU)214 mutation that disrupts the original termination codon in the +1 frame and shifts translation termination of this frame downstream, producing a 27.24 kDa product (Figure 7D). The levels of the arrest products derived from the *HA-SDD1 L214L-V5-FLAG* mRNA in *mbf1*Δ*ltn1*Δ mutant cells (Figure 7E, lane 8) were essentially the same as in *ltn1*Δ mutant cells (Figure 7E, lane 2), indicating that Mbf1 is dispensable for RQC induced by the *SDD1* staller. Importantly, the +1 RFS product was detectable in *mbf1*Δ mutant cells and *mbf1*Δ*ltn1*Δ mutant cells (Figure 7E, lanes 7 and 8), indicating that +1 RFS was induced by the *SDD1* stalling sequence. However, the 5′ NGD-IM product derived from *HA-SDD1 L214L-V5-FLAG* mRNA was almost eliminated in *mbf1*Δ*ski2*Δ mutant cells (Figure 7F, lane 4), confirming the essential role of Mbf1 in the NGD^RQC+^ by the *SDD1* staller.

We then examined the NGD and RQC by the endogenous staller *SDD1* in the Mbf1 mutants. The 5′ NGD-IM product derived from *HA-SDD-V5-FLAG* mRNA detected in Mbf1 wild-type cells (Figure 7G, lanes 2) was diminished in Mbf1 mutant cells (Figure 7G, lane 3–5), indicating a role for the Mbf1 interaction with uS3 in the NGD^RQC+^. We also introduced mutations in residues of Mbf1 that contribute to the interaction with mRNA in the colliding ribosomes (25). The association of Mbf1 mutants was not affected. The ribosome association of Mbf1 was diminished in Mbf1–K46A/K47A/Y48A/D66R quadruple mutant cells (Figure 7I), suggesting that the Mbf1–mRNA interaction strongly contributes to the ribosome association of Mbf1. The 5′ NGD-IM product derived from the *HA-SDD1 L214L-V5-FLAG* mRNA was detected in Mbf1 wild-type cells (Figure 7J, lane 2) but was significantly decreased in Mbf1–K46A/K47A/Y48A triple mutant cells (Figure 7J, lane 3) and in Mbf1–K46A/K47A/Y48A/D66R quadruple mutant cells (Figure 7J, lane 5), indicating a role for the Mbf1 interaction with mRNA in the NGD^RQC+^. RQC induced by the *SDD1* staller was not affected in the *mbf1*Δ*ltn1*Δ mutant cells expressing the indicated Mbf1 mutants (Figure 7K). To further verify the role of the Mbf1–uS3 interaction in NGD, we examined the RQC and NGD by the *SDD1* staller in the *uS3-K108E* mutant cells in which reading frame maintenance is defective (35). The *uS3-K108E* mutation disrupted the cleavage of the *SDD1* staller mRNA as no 5′ NGD-IM was detected in *ski2*Δ*uS3-K108E* cells (Supplementary Figure S3A, lane 6), although the arrest product was detected in *ltn1*Δ*uS3-K108E* (Supplementary Figure S3B, lane 12). Together, these results show that the interaction of Mbf1 with uS3 and mRNA in the colliding ribosome is required for NGD^RQC+^ induced by the endogenous *SDD1* staller mRNA.

## DISCUSSION

Ribosome stalling triggers quality control pathways targeting the mRNA (NGD) and the nascent polypeptide (RQC) in yeast. These two quality control pathways are coupled via Hel2 and both respond to ribosome collision. Recent studies demonstrated that a collided ribosomes consisting of the first stalled ribosome and the following colliding ribosome are the minimal ribosome collision unit, which is required to initiate both NGD and RQC (13). The endonucleolytic cleavage of an NGD reporter mRNA can occur either at sites within the collided ribosomes unit or upstream of it. The dual role of Hel2 drives these two distinct NGD branches, the NGD^RQC+^ and the NGD^RQC−^, which require specific ubiquitination events on the stalled collided ribosomes. In the NGD^RQC+^ pathway, Hel2-mediated ubiquitination of uS10 followed by the RQT complex-mediated subunit dissociation leads to cleavage events in the mRNA covered by the collided ribosomes unit. In the NGD^RQC−^ pathway, Hel2 ubiquitinates monoubiquitinated eS7 and plays a crucial role in the endonucleolytic mRNA cleavages that occur upstream of the stalled collided ribosomes (13).

Our results in this study present how Cue2 recognizes the substrates of NGD^RQC+^ and NGD^RQC−^ pathways in the rare codon-induced and in the endogenous *SDD1* mRNA-induced ribosome collision situation. We found that the N-terminal ubiquitin-binding CUE-D1 and CUE-D2 domains of Cue2 are required for the NGD^RQC−^ mRNA cleavage upstream of ribosomes collided on both the rare codons (Figure 2) and the endogenous *SDD1* stallers (Figure 4). Since the polyubiquitinated eS7 is a crucial modification for the cleavage upstream of the collided ribosomes (13), we propose that Cue2 is recruited to the polyubiquitinated eS7 via these CUE-D1 and CUE-D2 domains. One possibility is that CUE-D1 and CUE-D2 domains simultaneously associate with the polyubiquitinated eS7 of the colliding ribosomes on the rare codon and the *SDD1* staller mRNAs. In the case of the NGD^RQC+^ pathway, CUE-D1 and CUE-D2 domains are dispensable (Figures 1 and 2), but the W122 residue of Cue2 is critically required in both the rare codon (Figure 3) and the *SDD1* staller mRNAs (Figure 4). In NGD^RQC+^, the ubiquitinated 40S derived from the leading stalled ribosome was detected as a product of the RQT complex-mediated subunit dissociation, indicating that the RQT complex recognizes the polyubiquitinated uS10 of the first leading ribosome for subunit dissociation (12). Hel2-mediated K63-linked polyubiquitination and the Slh1-mediated subunit dissociation are both required for the endonucleolytic cleavage within the collided ribosome in both the rare codon staller (Figure 3) (13) and the endogenous *SDD1* staller mRNAs (Figure 4).

In this study, we investigated the role of Mbf1 in RQC and NGD induced by collided ribosomes stalled on various staller mRNAs including the endogenous *SDD1*, poly(A) and the *R(CGN)_12_* rare codons staller. The ribosome binding of Mbf1 is essential for both the RQC and the NGD induced by the *R(CGN)_12_* rare codons staller (Figure 5). Both RQC and NGD were decreased in uS3 interaction-defective Mbf1 mutants (Figure 5F, G). Importantly, the efficiency of +1 RFS (Figure 5J, lanes 2–5) was highly correlative with that of the reduction of NGD (Figure 5F, lanes 2–5) and RQC products (Figure 5G, lanes 6, 8 and 10). The level of +1 RFS products derived from *the GFP-R(CGA)_6_–2HA-+G-FLAG-HIS3* reporter was also increased in the Mbf1 mutants that are defective in the ribosome association (Figure 5E, J), suggesting that the maintenance of the reading frame by Mbf1 is required for RQC and NGD by the rare codons staller.

In contrast to the *R(CGN)_12_* rare codon staller, both RQC and NGD induced by the poly(A) staller are intact in the *mbf1*Δ and the *uS3-K108E* mutant cells (Figure 6). This suggests that Mbf1 is not required for NGD or RQC induced by translation of poly(A) tracts in yeast. Since previous reports indicate that poly(A) tracts induce +1 RFS in yeast (28), we suspect that +1 RFS is induced in *mbf1* mutant cells but does not change the sense of the poly(A) sequence and induces quality controls. We investigated the role of Mbf1 in the repression of +1 RFS, RQC and NGD induced by the poly(A) staller. The +1 RFS product derived from *GFP-K(AAA)_12_-+G-FLAG-HIS3* was not detected in the *hel2*Δ and *mbf1*Δ mutant cells (Figure 6C, lanes 2 and 4), indicating that Mbf1 may repress the +1 RFS induced by the poly(A) staller. One possibility is that the sense and stalling mechanism of the translated poly(A) tract does not change after the +1 RFS and therefore results in a notably lower amount of the +1 RFS product compared with the rare codons staller (Figure 5I). Interestingly, Mbf1 was suggested to specifically interact with the second colliding ribosome in a hybrid state (25,26). However, ribosomes collided on a poly(A) stretch were reported to largely adopt a POST–POST state collision conformation (34), which would not provide the suitable hybrid state ribosome for Mbf1 interaction. Therefore, the specific ribosome collision conformation induced by the poly(A) stalling could prevent Mbf1 interaction with the colliding ribosome(s) and potentially rely on other factors. Taken together, the architecture of eukaryotic ribosome collisions is not uniform and we demonstrate that the requirement of Mbf1 for quality control pathways depends on the particular stalling sequence. The mode of Mbf1–mRNA interactions may be different in the collided ribosomes induced by rare codons, poly(A) and *SDD1* stallers, and the differences in the defect in NGD by the Mbf1 mutants may be reflected by the specificity of the interaction.

Our results indicate that Mbf1 is required for the NGD^RQC+^ cleavage at the colliding ribosome of the *SDD1* staller (Figure 7). The T69, D70, I71 and V72 residues of Mbf1 are crucial for the binding to the uS3 (Figure 5). The level of the arrest protein products derived from the *HA-SDD1-V5-FLAG* reporter in the *ltn1*Δ background was not reduced in the Mbf1–T69A/D70A/I71A/V72A mutant cells (Figure 7H), but the level of the 5′ NGD-IM products derived from the *HA-SDD1-V5-FLAG* reporter in the *ski2*Δ background was reduced (Figure 7G). This indicates that the ribosome binding of Mbf1 is crucial for the NGD^RQC+^ but not for the RQC induced by the *SDD1* staller. We noticed that, in the Mbf1-D66R mutant cells, the level of 5′ NGD-IMs derived from the *GFP-R(CGN)_12_-FLAG-HIS3* mRNA was decreased (Figure 5F, lane 3). The +1 RFS by the rare codons staller was increased in the Mbf1-D66R mutant cells, indicating that D66R mutation moderately affects Mbf1 function in the repression of +1 RFS by the rare codons staller. Taken together, the +1 RFS efficiency estimated with the level of the +1 RFS product derived from the (CGA)_6_ staller reporter (Figure 5J) in the Mbf1 mutants is consistent with that of the defects of NGD by the rare codon staller (Figure 5F) and *SDD1* staller (Figure 7G), strongly suggesting that the defects in NGD may be due to the increase of the +1 RFS. We propose that Mbf1 represses +1 RFS during ribosome stalling to induce quality controls. The NGD^RQC+^ efficiency is more sensitive to the defects in the maintenance of the reading frame than that of RQC, suggesting that Mbf1 must prevent +1 RFS to maintain the specific collided ribosomes for the Cue2 recruitment after RQT-mediated splitting of the collided ribosomes.

**Figure 8.**
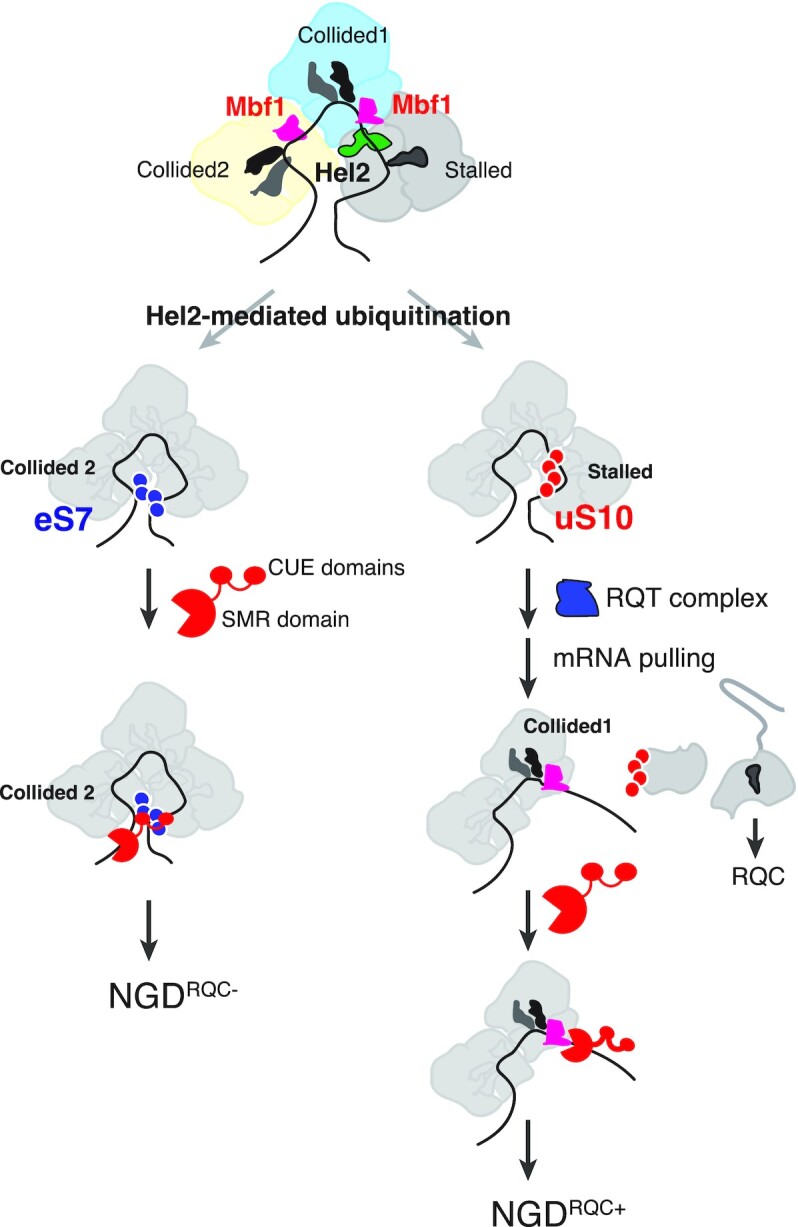
A model for the role of Cue2 in two modes of NGD. A model for Cue2 recognition of the substrates of two modes of NGD induced by the rare codon staller. A model for NGD^RQC−^ (left): Hel2 forms K63-linked polyubiquitin chains on eS7 (shown in blue circles) of the colliding ribosome in the trisome formed on the *SDD1* mRNA. Cue2 binds to the K63-linked polyubiquitin chains on eS7 with two CUE domains, CUE-D1 and CUE-D2. The Cue2 bound to the stalled ribosome cleaves the mRNA upstream of the colliding ribosome 2 (collided 2). A model for NGD^RQC+^ (right): Hel2 ubiquitinates uS10 on the leading ribosome and the RQT complex recognizes the polyubiquitinated uS10 (shown in red circles). The Slh1 helicase subunit of RQT applies a pulling force on the mRNA, leading to the dissociation of the leading ribosome into subunits. The collided ribosome 1 (collided 1) functions as a ram or giant wedge, but mRNA is partially released. After pulling of mRNA by the RQT complex, Cue2 cleaves mRNA partially released from the colliding ribosome 1.

Finally, we propose a model of Cue2 activity induced in NGD (Figure 8). As an initial step of NGD^RQC−^ (left branch in Figure 8), Hel2 forms K63-linked polyubiquitin chains on eS7 of the colliding ribosome(s). Subsequently, Cue2 binds to the K63-linked polyubiquitin chains on eS7 with the first two N-terminal CUE domains (CUE-D1 and CUE-D2). The Cue2 bound to the colliding ribosome then cleaves the mRNA upstream of the colliding ribosome. As an essential step for NGD^RQC+^ (right branch in Figure 8), Hel2 ubiquitinates uS10 on the leading ribosome (shown in red circles), which is a mark recognized by the RQT complex. The RQT complex then dissociates the leading ribosome into subunits. The Slh1 helicase of the RQT complex may modulate the structure of the second colliding ribosome for Cue2 to access the cleavage site within the colliding ribosome. Slh1 is a Ski2-like RNA helicase and is therefore likely to rearrange RNA structure and/or RNA–protein interactions in an ATP-dependent manner. Slh1 may also pull on the mRNA to allow Cue2 to access the cleavage site in the colliding ribosome. Recent cryo-EM analysis of several RQT–ribosome complexes revealed the structural basis of splitting (36). We propose that RQT engages the 40S subunit of the lead ribosome and can switch between two conformations. The Slh1RNA helicase subunit of RQT applies a pulling force on the mRNA, causing destabilizing conformational changes in the 40S subunit. The collided ribosome functions as a giant wedge, ultimately resulting in subunit dissociation. We suspect that, after the pulling of mRNA by the RQT complex, mRNA is also partially released from the collided ribosome and is cleaved by Cue2. In the NGD^RQC+^ by the *SDD1* staller, Mbf1 is required for the Cue2-mediated cleavage at the sites in the colliding ribosome (Figure 7A) but not for the splitting of the stalled ribosome that is required for RQC (Figure 7H, K). Based on the model for the RQT-mediated splitting by the pulling of mRNA, Mbf1 may contribute to the recruitment of Cue2 at the sites located within the colliding ribosome after the mRNA pulling by the RQT complex. Further analysis of the mechanism by which the RQT complex mediates the subunit dissociation may provide the mechanistic insight for the NGD^RQC+^ by Cue2. Another possibility is that 40S subunits remain associated with mRNA after the subunit dissociation reaction and could represent a specific substrate for the mRNA cleavage by Cue2. It is important to emphasize that, regardless of the proposed pathway, the W122 residue critically contributes to the Cue2-mediated NGD^RQC+^ cleavage, and Mbf1 should play a crucial role in the recruitment of Cue2 by the *SDD1* staller. It will be interesting to reveal the exact molecular basis by which Cue2 activity is coordinated for the mRNA cleavage by the presence of distinct ubiquitin patterns, Mbf1 and RQT-mediated subunit dissociation.

The ubiquitination sites of ribosome proteins and their specific roles in quality control and gene expression have been identified and demonstrated. The key question is how the specific reader of the ubiquitin chain on ribosome proteins induces quality control and expression regulation. The RQT complex recognizes polyubiquitinated uS10 and dissociates the stalled ribosome in the collided ribosomes (12). Two-step ubiquitination of uS3 is crucial for the rapid decay of the non-functional 80S ribosome with the mutation in the 18S decoding center (37). Based on the results shown in this study, we propose that Cue2 binds to the polyubiquitinated eS7 and cleaves mRNA in the vicinity of collided ribosomes. Further experiments will reveal how Cue2 specifically recognizes the polyubiquitinated eS7.

## DATA AVAILABILITY

The data underlying this article are available in Mendeley at https://data.mendeley.com/datasets/4pc5bp4c48/1.

## Supplementary Material

gkac1172_Supplemental_Files
