# Supplementary material for: Two modes of Cue2-mediated mRNA cleavage with distinct substrate recognition initiate no-go decay
Source: Nucleic Acids Res. 2022 Dec 30;51(1):253–70. doi: 10.1093/nar/gkac1172 (PMC9841427; doi:10.1093/nar/gkac1172)
Supplement: gkac1172_Supplemental_Files [file gkac1172_supplemental_files.zip › NAR-01139-X-2022 Supplementary information 1003.pdf]

Supplementary Information

Two modes of Cue2-mediated mRNA cleavage with distinct substrate recognition initiate No-go decay

Shota Tomomatsu[1,2,3,#], Atsuya Watanabe[3,#], Petr Tesina[4,] Satoshi Hashimoto[3], Ken Ikeuchi[3,4], Sihan Li[1,3], Yoshitaka Matsuo[1,3], Roland Beckmann[4], Toshifumi Inada[1,3*]

[1]Division of RNA and gene regulation, Institute of Medical Science, The University of Tokyo, Minato-Ku 108-8639, Japan,[2]Graduate School of Pharmaceutical Sciences, The University of Tokyo, Bunkyo-Ku, Tokyo, Japan, [3]Graduate School of Pharmaceutical Sciences, Tohoku University, Sendai 980-8578, Japan, [4]Gene Center and Department of Biochemistry, Feodor-Lynen-Str. 25, University of Munich, 81377 Munich, Germany

[#]These authors contributed equally to this study.

Key words: Colliding ribosome; No-go mRNA decay (NGD); ribosome quality control (RQC); ribosome ubiquitination; RQT complex; Cue2; Mbf1

*Corresponding author
Prof. Dr. Toshifumi Inada. Email: toshiinada@ims.u-tokyo.ac.jp (T.I.)

Lead contact:
To whom correspondence should be addressed: Toshifumi Inada, Minato-Ku 108-8639, Tokyo, Japan. Tel: +81 (3)5449-5275, Fax: +81 (3)5449-5275, E-mail: toshiinada@ims.u-tokyo.ac.jp (T.I.)

Running title: Two modes Cue2-mediated cleavages in NGD

This PDF file includes:
Supplementary Figures 1 to 3
Supplementary Tables 1 to 2

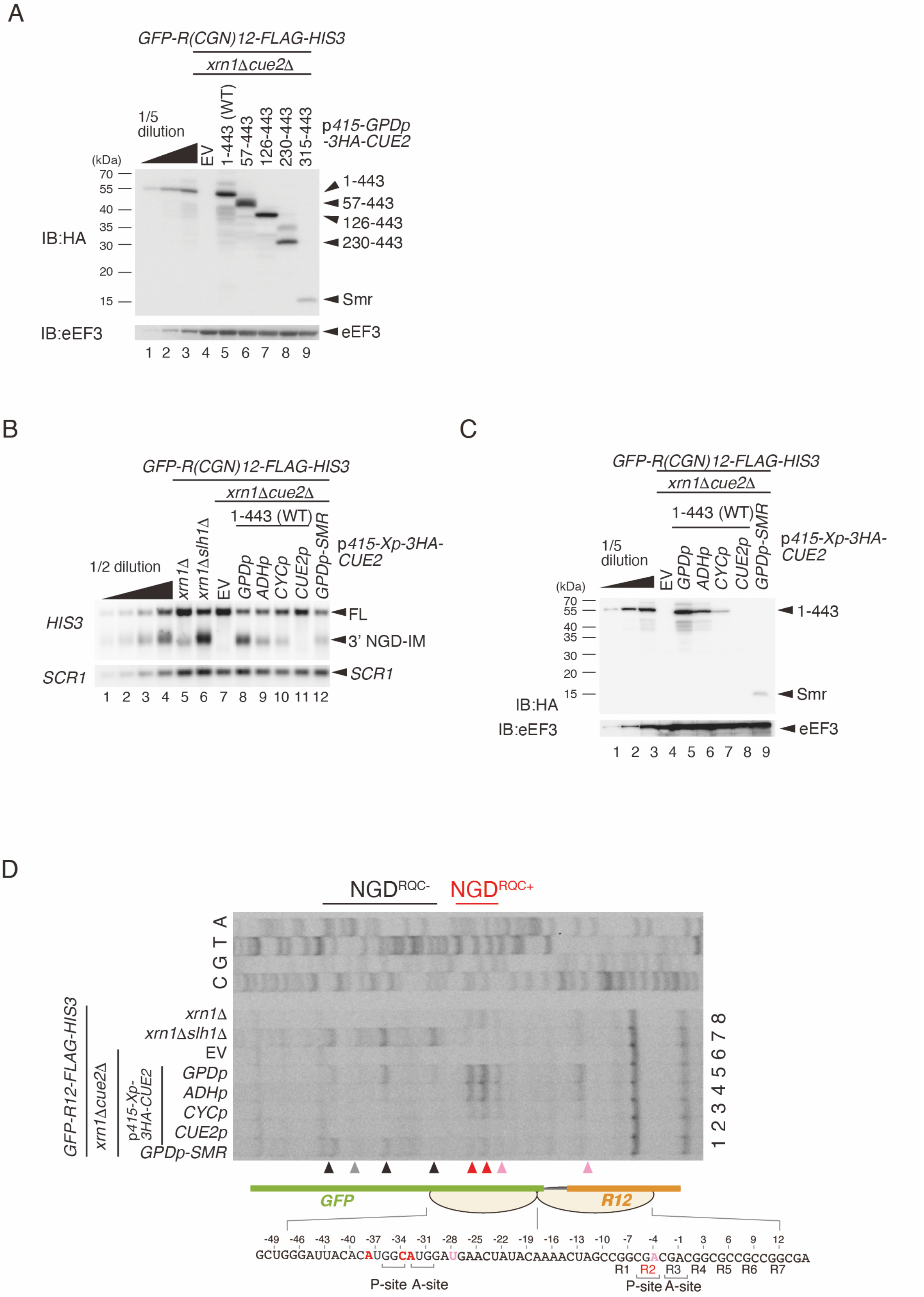

Figure S1. The efficiency of NGD is increased together with the expression level of Cue2.

(**A)** Western blot analysis to check the expression levels of HA-tagged Cue2 deletion mutant proteins. **(B)** Northern blot analysis of NGD-cleavage sites in cells expressing various levels of Cue2. The full-length *GFP-R(CGN)$_{12}$-FLAG-HIS3* mRNA and 5' NGD-intermediates (5' NGD-IM) or 3' NGD-intermediates (3' NGD-IM) were detected in the indicated mutant cells by Northern blotting with DIG-labelled probes. 3' NGD-intermediates were detected by the DIG-labelled *HIS3* probe. *SCR1* was used as a loading control. FL = full-length. **(C)** Western blot analysis to check the expression levels of HA-tagged Cue2 deletion mutant proteins as schematically displayed in (A) using an anti-HA antibody. **(D)** Primer extension mapping of 5' ends of 3' NGD-intermediates in cells expressing various levels of Cue2. The primer extension samples were analyzed using 5% TBE-Urea-PAGE and detected by fluorescence. Non-specific reverse transcription (ReTr) products are indicated by asterisks. The red arrowheads indicate the Cue2-mediated cleavage sites in the colliding ribosome in the presence of Slh1, and the black arrowheads indicate Cue2-mediated mRNA cleavage sites upstream of the colliding ribosome without the subunit dissociation by Slh1.

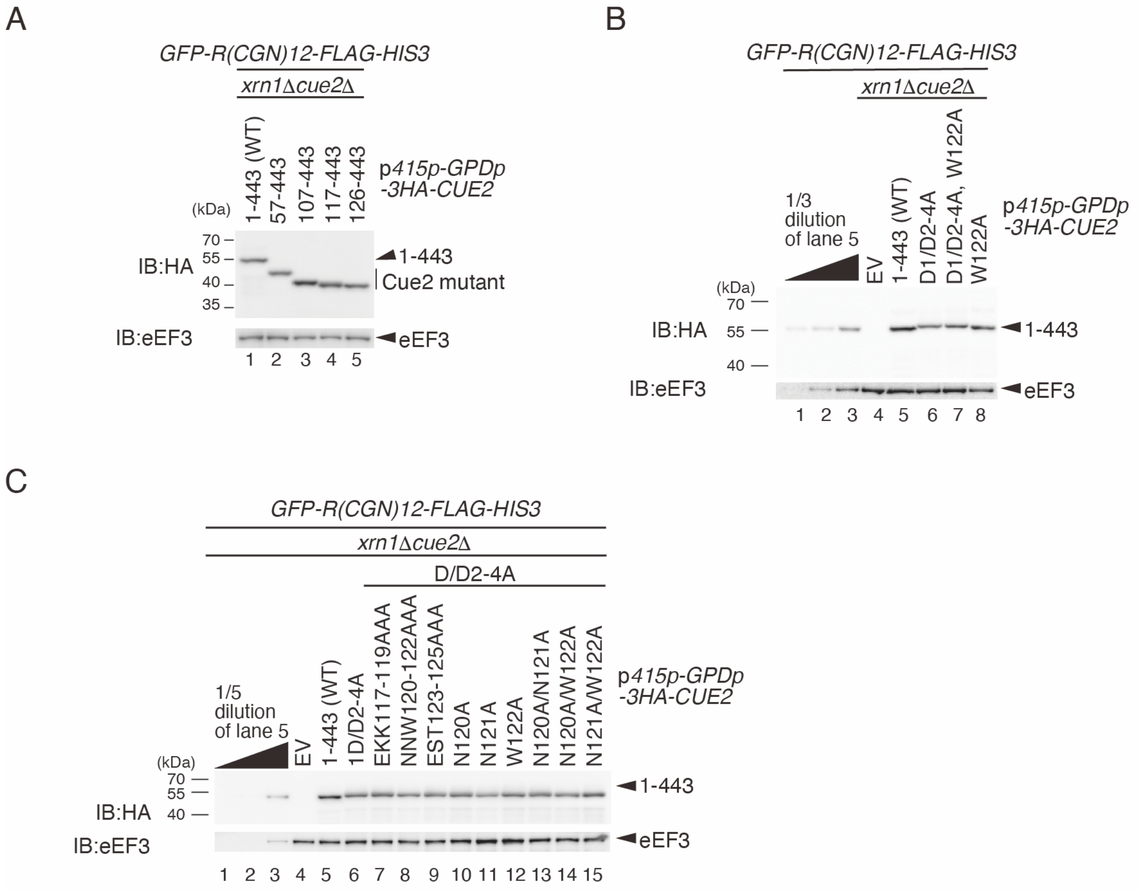

Figure S2. The expression level of Cue2 deletion mutants.

(A-C) Western blot analysis to check the expression levels of HA-tagged Cue2 deletion mutant proteins as schematically displayed in Fig. 3A **(A)**, Fig 3C **(B)**, and Fig. 3D **(C)** using an anti-HA antibody.

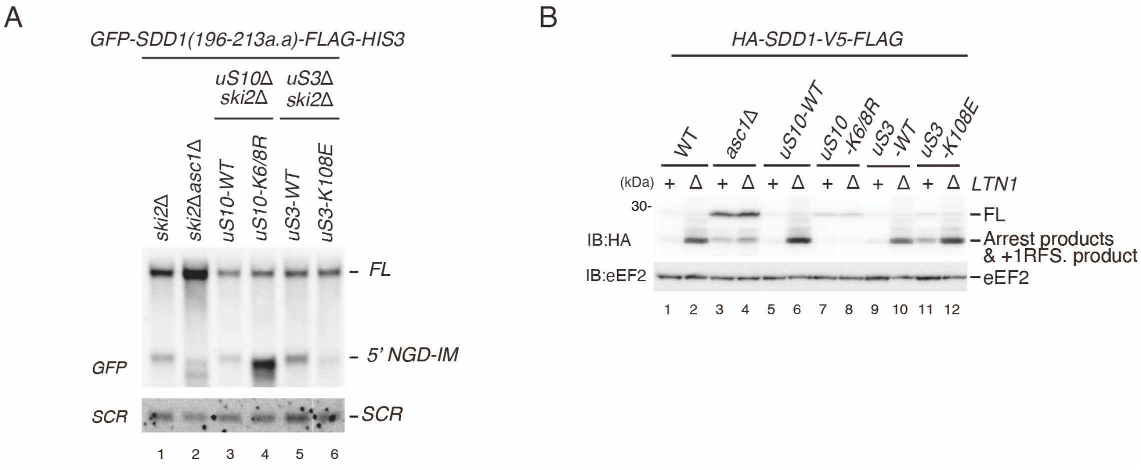

Figure S3. The K108 of uS3 is required for NGD but not for RQC induced by the *SDD1* staller.

(A) The K108 of uS3 is crucial for NGD induced by the SDD1 staller. 5′-NGD-IMs derived from the *GFP-SDD1(196-213a.a.)-FLAG-HIS3* reporter was detected in the W303*ski2*Δ, *asc1*Δ*ski2*Δ, *uS10-K6/8R ski2*Δ, *uS3-WTski2*Δ or *uS3-K108Eski2*Δ mutant cells by northern blotting with a DIG-labelled *GFP* or *SCR1* probe. **(B)** Western blot suggesting that RQC induced by *SDD1* is intact in *uS3-K108* mutant cells. The arrest products in the *ltn1*Δ background derived from the *HA-SDD1-V5-FLAG* were detected in the W303, *asc1*Δ, *uS10-K6/8R, uS3-WT,* or *uS3-K108E* mutant cells with an anti-HA antibody.
